# “One Method to Label Them All”: A Single Fully Automated Protocol for GMP-Compliant ^68^Ga Radiolabeling of PSMA-11, Transposable to PSMA-I&T and PSMA-617

**DOI:** 10.2174/0118744710293461240219111852

**Published:** 2024-02-28

**Authors:** Juliette Fouillet, Charlotte Donzé, Emmanuel Deshayes, Lore Santoro, Léa Rubira, Cyril Fersing

**Affiliations:** 1 Nuclear Medicine Department, Institut Régional du Cancer de Montpellier (ICM), University of Montpellier , Montpellier, France;; 2 Institut de Recherche en Cancérologie de Montpellier (IRCM), INSERM U1194,University of Montpellier, Montpellier, France;; 3 IBMM, University of Montpellier, CNRS, ENSCM, Montpellier, France

**Keywords:** PET imaging, prostate cancer, PSMA-11, PSMA-617, PSMA-I&T, ^68^Ga, automated synthesis, radiopharmacy

## Abstract

**Background:**

Prostate-specific membrane antigen (PSMA) is an ideal target for molecular imaging and targeted radionuclide therapy in prostate cancer. Consequently, various PSMA ligands were developed. Some of these molecules are functionalized with a chelator that can host radiometals, such as ^68^Ga for PET imaging. The ^68^Ga radiolabeling step benefits from process automation, making it more robust and reducing radiation exposure.

**Objective:**

To design a single automated radiolabeling protocol for the GMP-compliant preparation of [^68^Ga]Ga-PSMA-11, transposable to the production of [^68^Ga]Ga-PSMA-617 and [^68^Ga]Ga-PSMA-I&T.

**Methods:**

A GAIA^®^ synthesis module and a GALLIAD^®^ generator were used. Radio-TLC and radio-HPLC methods were validated for radiochemical purity (RCP) determination. Three [^68^Ga]Ga-PSMA-11 validation batches were produced and thoroughly tested for appearance and pH, radionuclide identity and purity, RCP, stability, residual solvent and sterility. Minimal modifications were made to the reagents and disposables for optimal application to other PSMA ligands.

**Results:**

[^68^Ga]Ga-PSMA-11 for clinical application was produced in 27 min. The 3 validation batches met the quality criteria expected by the European Pharmacopoeia to allow routine production. For optimal transposition to PSMA-617, the solid phase extraction cartridge was changed to improve purification of the radiolabeled product. For application to PSMA-I&T, the buffer solution initially used was replaced by HEPES 2.7 M to achieve good radiochemical yields. Residual HEPES content was checked in the final product and was below the Ph. Eur. threshold.

**Conclusion:**

A single automated radiolabeling method on the GAIA^®^ module was developed and implemented for ^68^Ga radiolabeling of 3 PSMA ligands, with slight adjustments for each molecule.

## 1 INTRODUCTION

**Fig. (1):**
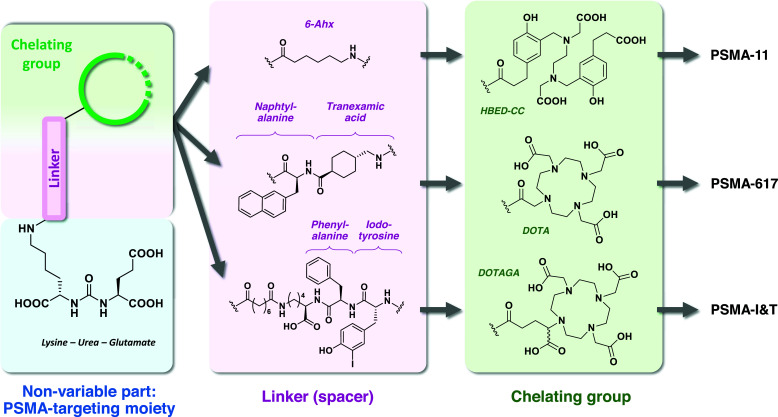
Comparison of the chemical structure of the linker and chelator moieties found in ^68^Ga-radiolabelled PSMA ligands used as PET imaging agents.

First described in the late 1980s [1], prostate-specific membrane antigen (PSMA) is a type II transmembrane glycoprotein found on cells surface as a monomer or homodimer [2-4]. Its structure consists of a short cytoplasmic N-terminal region, a 24-amino acid transmembrane domain, and a 707-amino acid extracellular C-terminal region [5-7]. Early studies on brain cells PSMA highlighted an N-acetylated-alpha-linked acidic dipeptidase (NAALDase) activity [2]. Also known as folate hydrolase 1 (FOLH1) or glutamate carboxypeptidase II (GCPII), PSMA plays a role in folates and glutamate metabolism by releasing glutamic acid from folate polyglutamate derivatives and catalyzing the hydrolysis of N-acetylaspartylglutamate (NAAG) into glutamate and N-acetyl aspartate. In prostate cancer, PSMA-expressing cells consequently display increased folate uptake and rapid proliferation [8-10], as PSMA-catalyzed glutamate release activates phospholipase C pathway and promotes tumor growth [11, 12]. In prostate cancer cells, PSMA expression levels are approximately a thousand-fold higher compared to normal prostate tissues, with the highest levels observed in poorly differentiated and castration-resistant tumor cells [13, 14]. Furthermore, only minimal PSMA expression is found in other organs such as proximal kidney tubules, brain, lacrimal and salivary glands, peripheral ganglia, breast tissue, and duodenal brush border epithelium [13, 15, 16]. Accordingly, PSMA represents an ideal target for the specific delivery of molecular imaging or targeted therapy agents to prostate cancer cells. In this perspective, small molecules with high affinity and specificity for PSMA were designed to bind this target, in particular for diagnostic and therapy applications in nuclear medicine [17]. Regarding their chemical structure, all PSMA ligands for clinical purposes are composed of 3 parts (Fig. **1**). First, a non-variable PSMA-binding motif is formed of a pentanedioic acid acting as glutamate mimic, a urea moiety acting as a complexing group for a zinc atom located in the catalytic site of the enzyme [18, 19], and a lysine residue to obtain an easily functionalizable pseudo-dipeptide. Then, a linker (or spacer) part is used to introduce distance between the Glutamate-Urea-Lysine (KuE) scaffold and the radiolabel-bearing moiety. Most importantly, the linker tends to modulate the overall physicochemical and pharmacokinetic properties of the molecule [20–23]. For example, while a simple 6-aminohexanoic acid (Ahx) chain is used in PSMA-11 [24], a naphtylalanine/tranexamic acid group is introduced in PSMA-617 to enhance tumor uptake and reduce renal tracer accumulation [25, 26]. Similarly, the longer linker of PSMA-I&T (PSMA-1) containing both phenylalanine and iodotyrosine contributes to the higher internalization capacity of the molecule [27, 28]. Finally, a chemical group suitable for bearing a radioactive atom functionalizes these constructs. This includes dedicated prosthetic groups to accommodate non-metallic radioelements (*e.g*. ^18^F, carried by a 6-[^18^F]fluoronicotinic acid prosthetic group in [^18^F]DCFPyL [29]) and chelating moieties for radiometals complexation. For diagnostic applications, ^68^Ga emerged as an ideal radioelement for PET imaging, owing to its availability *via* a ^68^Ge/^68^Ga generator and its straightforward, well-mastered radiochemical characteristics [30]. For the complexation of ^68^Ga, linear chelators such as HBED-CC are widely adopted, as they allow rapid complexation and low-demand radiolabeling conditions [21, 31-33]. Featured in PSMA-11 [21], HBED-CC is especially suited to ^68^Ga, but is incompatible with other radiometals. Consequently, cyclic chelating agents are more likely to be used for theranostic applications. Although such complexing agents are more demanding regarding radiolabeling conditions, they are also more versatile and accommodate a wide variety of radiometals. Thus, a DOTA chelator was chosen in PSMA-617, allowing complexation with both ^68^Ga for PET imaging and beta-minus emitter ^177^Lu, or even alpha emitter ^225^Ac for targeted radionuclide therapy [34]. Similarly, a DOTAGA chelator derived from DOTA was introduced in PSMA-I&T, enabling theranostic use and enhancing the overall hydrophilicity of the molecule [27]. To date, PSMA-11 stands out as the most widely used PSMA ligand for ^68^Ga PET imaging of prostate cancer, however, PSMA-617 [35-43] and PSMA-I&T [44-52] can also be radiolabeled with ^68^Ga and used in clinical settings, mostly through research protocols.

To ensure a reliable, robust, and good manufacturing process (GMP)-compliant preparation procedure, the in-house radiolabeling of molecules such as PSMA ligands benefits from automation, with the growing use of synthesis modules [53]. A series of models is commercially available, most of them relying on single-use tubing sets and dedicated reagent kits. So far, the radiosynthesis of [^68^Ga]Ga-PSMA-11 was reported in detail on Scintomics GRP [54, 55], GE TRACERlab FX_FN_ [56, 57], Trasis miniAiO [58, 59], Eckert & Ziegler Modular-Lab PharmTracer [60] and iPHASE MultiSyn [61] modules, but was not extensively described on the GAIA^®^ automaton (Elysia-Raytest) [62]. Thus, we present herein a comprehensive report on the development and validation of all the protocols for the automated, GMP-compliant ^68^Ga radiolabeling of PSMA-11 on a GAIA^®^ synthesis module and for the corresponding quality controls (QCs). In addition, the applicability of this automated radiolabeling process to other PSMA ligands was studied with the preparation of [^68^Ga]Ga-PSMA-617 and [^68^Ga]Ga-PSMA-I&T.

## 2 MATERIALS AND METHODS

### 2.1 Reagent and Devices

GMP-grade PSMA-11 and [^nat^Ga]Ga-PSMA-11 reference standards were purchased from ABX pharmaceuticals (Advanced Biochemical Compounds, Germany) as lyophilized products in aliquots of 10 µg and 500 µg per vial, respectively. Sterile, single-use fluidic labelling kits for ^68^Ga labeling (reference RT-01-H) and reagent kits (reference RT-101) were also purchased from ABX Pharmaceuticals. A reagent kit included SCX (Bond Elut 100 mg with appropriate Luer adapter cap) and C_18_ cartridges (Sep-Pak^®^ Plus 360 mg sorbent), ammonium acetate buffer 0.08 M pH 4.6 (~4 mL), isotonic saline 0.9% (~8.6 mL), ethanol 60% (1.5 mL) and absolute ethanol (5 mL) vials, sodium chloride 5 M pH 1 (0.5 mL) syringe, water for injections (WFI, 100 mL) and 0.22 µm filter (Minisart^®^ 0.22 µm, 13 mm). ^68^Ga was obtained by elution of a pharmaceutical-grade ^68^Ge/^68^Ga generator (GALLIAD^®^ 1850 MBq, IRE Elit, Belgium) with ~1.1 mL of a 0.1 M HCl solution. The automated radiosynthesis of ^68^Ga-labeled PSMA ligands was conducted on a GAIA^®^ synthesis module (Elysia-Raytest, Germany) driven by the appropriate software (GAIA control, Elysia-Raytest, Germany). Production was conducted within the radiopharmaceuticals preparation unit (GMP grade C clean room) in a grade A shielded cell with a laminar airflow (MEDI 9000 Research 4R, LemerPax, France), with both the automated synthesis module and the ^68^Ge/^68^Ga generator placed in the hot cell. Radioactivity in the product vial and in patient doses was measured in a calibrated ionization chamber (CRC^®^-25R, Capintec, USA).

### 2.2 Detailed Production Process of [^68^Ga]Ga-PSMA-11

**Fig. (2):**
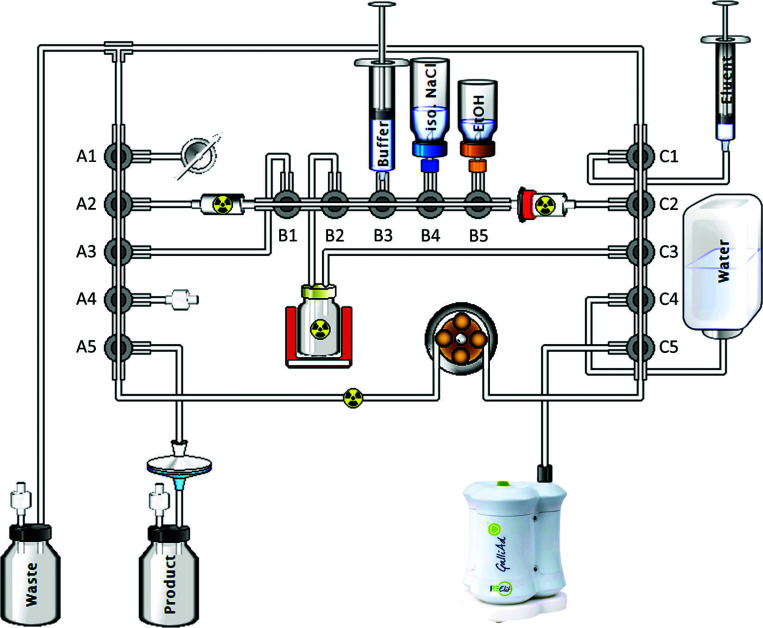
Configuration of the GAIA^®^ module for the synthesis of [^68^Ga]Ga-PSMA-11. The ramps are identified A, B and C and the valves are numbered from 1 to 5 on each ramp.

Before starting the synthesis, the C_18_ cartridge was properly preconditioned with 5 mL absolute ethanol and 5 mL WFI, then the single-use kit was set up with reagents on the automaton (Fig. **2**). After initialization of the customized synthesis protocol, the module performed a kit integrity test to prevent any leakage during preparation. Then, PSMA-11 solubilized in 2.8 mL of ammonium acetate buffer solution was transferred to the reaction vial and the SPE cartridges were conditioned with WFI, before the tubing lines were purged with filtered air. The ^68^Ge/^68^Ga generator was subsequently eluted with 1.1 mL of 0.1 N HCl, the vacuum suction transferring the eluate through the SCX cartridge on which ^68^Ga was trapped. After peristaltic pumping to finish the elution process, the SCX cartridge was washed with WFI before the tubing lines were purged with filtered air. [^68^Ga]Ga^3+^ from the SCX cartridge was then eluted to the reaction vial with 0.5 mL of NaCl 5M pH 1. The radiolabeling reaction proceeded for a total of 8 minutes at a temperature of 97°C. The reaction medium was then transferred to the SPE cartridge, with subsequent rinsing of the reaction vial and tubing with ~10 mL WFI. Free [^68^Ga]Ga^3+^, not retained by the SPE cartridge, was removed to the waste vial through a washing step with WFI while [^68^Ga]Ga-PSMA-11 was trapped on the cartridge. The active substance was then eluted from the cartridge to the product vial by alternating fractions of 60% ethanol (1.5 mL total) and 0.9% NaCl (8.6 mL total). The majority of ^68^Ga colloids potentially formed during radiolabeling remained trapped on the cartridge. Sterilizing filtration was ensured by a 0.22 μ end filter and the integrity of the filter was finally checked by the module at the end of the preparation by a bubble point integrity test (minimum value set at 2.5 bar). The overall [^68^Ga]Ga-PSMA-11 radiosynthesis process on the GAIA^®^ module is summarized in Table **1** and the detailed automated sequence is provided in Supplementary materials.

To validate the preparation process of [^68^Ga]Ga-PSMA-11, three standardized validation batches were produced and subjected to extensive QCs. The definition of the product specifications was based on the Ph. Eur. monograph for [^68^Ga]Ga-PSMA-11 [63].

### 2.3 Radiochemical Yield Calculation

Throughout the automated synthesis process, the activities measured on the SCX cartridge and on the C_18_ cartridge were used to calculate radiochemical yields (RCY), expressed in %, according to the formula:


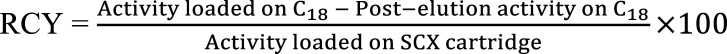


For routine production in clinical settings, a minimum total activity of 150 MBq at the end of the synthesis was set for the preparation of [^68^Ga]Ga-PSMA-11, approximately representing the average dose for a single patient of standard weight.

### 2.4 Quality Controls for [^68^Ga]Ga-PSMA-11

#### 2.4.1 Appearance and pH

The visual inspection of [^68^Ga]Ga-PSMA-11 preparations was performed right after the end of the synthesis to check the absence of particulates in the sample and the colorlessness of the solution [63].

The pH was checked on the final compounded [^68^Ga]Ga-PSMA-11 preparations by using 2-zones Rota pH 1 - 11 indicator paper (VWR, PA, USA) to ensure that this value is within the accepted range (4 to 8 [63]) according to the synthesis process and is appropriate for intravenous injection.

#### 2.4.2 Radionuclide Identity

A gamma-spectrometry analysis was conducted on a sample (~150 kBq) of each [^68^Ga]Ga-PSMA-11 validation batch using a Hidex AMG^®^ (LabLogic, UK) gamma counter. This analysis aimed to measure the emitted energies, with a special focus on the 511 keV and 1077 keV peaks from the annihilation photons, as specified by the European Pharmacopoeia [63, 64]. Half-life was determined by repeated measurements of each validation batch sample over ~4 h (6 to 8 measurements per batch), aiming for a result within the expected range of 61 to 75 minutes and with an anticipated value of 67.71 min [65, 66].

To validate the linearity of the gamma counter-response, successive dilutions of a ^68^Ga generator eluate were made to obtain a range of 16 standards between 430 kBq/mL and 13 Bq/mL. Radioactive concentration (in Bq/mL) was plotted with the gamma counter response (in CPM) normalized to the initial measurement time to determine the linear correlation coefficient R^2^.

#### 2.4.3 Radionuclide Purity

For radionuclide purity determination, the same validation batch samples used for radionuclide identity assays were measured for 120 min in the gamma spectrometer, after a 48-h decay and under the same geometric conditions. With these settings, the eventual activity of ^68^Ga resulting from a ^68^Ge breakthrough and any other radionuclide impurity with a long half-life (> 5 h) was measured. The total radioactivity measured after 48 h should not be more than 0.001% of the radioactivity initially measured in each sample [65].

#### 2.4.4 Analytical HPLC Procedures

Radio-high-performance liquid chromatography (HPLC) analyses were carried out on a Nexera X3 apparatus (Shimadzu, Japan) using HPLC-grade solvents. The radio-HPLC station consisted of a solvent degasser (DGU-405), a solvent pump (LC40D), an autosampler (SIL-40) set at 20 µL injection volume, a column oven (CTO-40S) set at 30°C, a UV detector (SPD-40 190-700 nm) set at 220 and 280 nm and a radioactivity detector (GABI Nova with mid-energy probe and 2 × 5 µL flow cell) connected in series. The stationary phase was a C_18_ ACE^®^ Equivalence™ column, 3.0 x 150 mm, 110 Å pore size and 3 μm particle size. The flow rate was 0.6 mL/min, and the mobile phase gradient was programmed from 0.1% TFA in water (**A**) to 0.1% TFA in acetonitrile (**B**) as follows: 0 – 0.5 min 95/5 A/B; 0.5 – 10 min linear gradient from 95/5 A/B to 60/40 A/B; 10 – 11 min linear gradient from 60/40 A/B to 95/5 A/B; 11 – 16 min 95/5 A/B. Radio-HPLC analyses were performed using the appropriate acquisition and analysis software (Gina Star 10, Elysia-Raytest, Germany).

##### 2.4.4.1 Radio-HPLC Method Validation

Validation of the radio-HPLC analytical method for RCP determination of [^68^Ga]Ga-PSMA-11 was conducted by adapting ICH Q2 (R1) guidelines to the analysis of radioactive compounds [67].

To determine the linearity of radio detection, considering the short physical half-life of ^68^Ga, successive analyses from the same [^68^Ga]Ga-PSMA-11 final product batch were carried out. The areas of the two isomer peaks were summed up to express the total area as a function of volume activity at the time of analysis. The coefficient of determination R^2^ was then calculated by linear regression. Thus, eleven HPLC measurements were performed between 21 min and 461 min after the end of synthesis on a [^68^Ga]Ga-PSMA-11 sample with an initial volume activity of 69.4 MBq/mL. The range of activity volumes used for linearity assays covered the range of values observed in clinical practice. A coefficient R^2^ ≥0.99 was expected.

Specificity was determined by analyzing in triplicate three independent [^68^Ga]Ga-PSMA-11 batches, containing all components that may be present at the time of the HPLC QC of the preparation. Impurities that could be detected included free ^68^Ga^3+^ (2 radio-HPLC peaks), ^68^Ga-containing impurities (4 radio-HPLC peaks) probably consisting of radiolysis products and possibly the third isomer of the PSMA-HBED-CC complex with gallium-68 [24]. As the HPLC peaks of the two main [^68^Ga]Ga-PSMA-11 isomers were close, the specificity of the method was considered sufficient for a resolution >1.5 between these two peaks and with surrounding signals.

Repeatability, as a part of precision, was determined by measuring the RCP of a [^68^Ga]Ga-PSMA-11 preparation, repeated 6 times under identical operating conditions and over a short time period. For each analysis, the area of each detected chemical species was corrected for decay to calculate the coefficient of variation (%CV) according to the formula: %CV = *s*/*m* × 100, where *s* is the standard deviation of the peak areas and *m* is the average of the peak areas. CV values ≤ 5.0% were expected for each chemical species.

To appreciate accuracy and exclude underestimation of impurities by irreversible retention on the stationary phase, the proportion of injected activity recovered at the column outlet was measured in triplicate over RCP analyses of a single [^68^Ga]Ga-PSMA-11 preparation. Aliquots of the final product and the post-column recuperation were counted in a gamma counter, decay corrected, and volume activities were compared to calculate recovery (%).

The limit of quantification (LOQ) of the radio-HPLC method was determined experimentally by successive analyses of samples from the same [^68^Ga]Ga-PSMA-11 preparation until the lowest volume activity with a signal-to-noise ratio >10 was reached. The LOQ was determined for both isomer 2 of [^68^Ga]Ga-PSMA-11 (characterized by a smaller peak than isomer 1) and for the smallest ^68^Ga^3+^ impurity peak.

The limit of detection (LOD) of the radio-HPLC method for isomer 2 of [^68^Ga]Ga-PSMA-11 and for ^68^Ga^3+^ impurity was determined in the same way as the LOQ until the lowest volume activity with a signal-to-noise ratio greater than 3 was reached.

To determine whether the method is capable of efficiently resolving close chromatographic peaks, and that no compound is co-eluted with another chemical species, a high-resolution test was carried out on 3 [^68^Ga]Ga-PSMA-11 batches. For this test, the gradient time set in the HPLC method studied (9.5 min) was doubled to improve compound separation. All other parameters remained unchanged.

##### 2.4.4.2 Chemical Identity Determined by HPLC

A reference solution was prepared by dissolving 500 μg of [^nat^Ga]Ga-PSMA-11 reference complex in 1 mL of a NaCl 0.9%/ethanol 60% (85/15) mixture. The reference solution was measured in triplicate under the HPLC conditions used for the radiochemical purity analysis of [^68^Ga]Ga-PSMA-11 (see 2.4.4.). A [^68^Ga]Ga-PSMA-11 preparation batch was also measured in triplicate and relative retention times (RRT) between [^nat^Ga]Ga-PSMA-11 and [^68^Ga]Ga-PSMA-11 were calculated according to the formula:


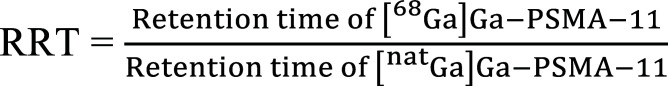


RRT had to be between 0.95 and 1.05 for the peak of each isomer.

##### 2.4.4.3 Radiochemical Purity Determined by HPLC

After synthesis, an aliquot of the [^68^Ga]Ga-PSMA-11 product was analyzed by radio-HPLC according to the previously described method for RCP determination. Considering the high sensitivity of the radio-HPLC technique, an RCP >91% was expected.

#### 2.4.5 Radiochemical Purity Determined by TLC

Radio-thin layer chromatography (TLC) analyses were performed using iTLC-SG strips (2 cm × 10 cm). A small drop of the [^68^Ga]Ga-PSMA-11 product was placed on a strip 2 cm above the baseline. The strip was then placed in a development chamber containing aqueous 1 M NH_4_OAc in methanol (1:1) as a mobile phase. The mobile phase was allowed to migrate approximately 1 cm below the top end of the strip. It was then removed from the chamber and placed into a TLC scanner (miniGITA^®^ Star, Elysia-Raytest, Germany) to determine the % areas of radioactivity at the origin and the solvent front, using the appropriate acquisition software (TLC Control v.2.30, Raytest, Germany) and analysis software (GINA Star TLC™ v.6.0, Elysia-Raytest, Germany). In these conditions, Rf (^68^Ga impurities) = 0.0–0.2 and Rf ([^68^Ga]Ga-PSMA-11) = 0.8–1.0. TLC analysis of a ^68^Ga solution at pH 1 (metal in the form of gallium (III) ions) and a gallium solution at pH 9 (metal in the form of colloids) was carried out in triplicate to confirm the Rf of ^68^Ga impurities (see Supplementary materials). It was also used to determine the LOQ of these impurities, measured at 5.65 kBq.

#### 2.4.6 Radiochemical Stability

The RCP of the three validation batches was measured by radio-TLC and radio-HPLC immediately after synthesis and then every hour up to 4 h after preparation. The final product was stored over this period at room temperature, at 24 ± 2°C.

#### 2.4.7 Adsorption in the Product Vial

A study was performed to prove the suitability of the product vial (TC-ELU-5, Curium, France), especially in terms of container-content interactions. From each [^68^Ga]Ga-PSMA-11 validation batch, 1 mL was withdrawn right after the end of synthesis and every hour up to 4 h. Each sample was measured in an ionization chamber, corrected for decay to the first measurement time, and the volumetric activities of each sample were compared. The coefficient of variation between the 4 volume activities of the same validation batch was expected <5%.

#### 2.4.8 Bacterial Endotoxins

The endotoxin level was determined by using a calibrated Endosafe^®^ nexgen-PTS^™^ unit (Charles River, MA, USA) that complies with Ph. Eur. 2.6.14. A limit of 175 endotoxin units (EU) per maximal injected dose is recommended; thus, for the 10.1 mL preparation of [^68^Ga]Ga-PSMA-11, the theoretical maximum limit would be 17.3 EU/mL. However, to guarantee the microbiological quality of the preparation, a slightly lower endotoxin limit was set at 12.5 EU/mL. The maximum significant dilution (MSD) of the [^68^Ga]Ga-PSMA-11 preparation to be tested was calculated according to the formula:


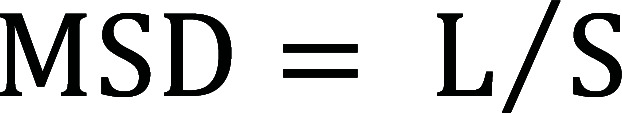


With L being the detection limit for endotoxins in the sample tested set at <1.0 EU/mL, and S being the intrinsic sensitivity of the technique, which is 0.05 EU/mL. This dilution ensures that the sample does not interfere with the UV measurement of the assay (% recovery between 50 and 200 and low %CV between measurements).

#### 2.4.9 Sterility Testing

Sterility tests were carried out on the three validation batches by direct inoculation method (Ph. Eur. 2.6.1 and 0125). Three 1 mL aliquots of the drug products were tested by a GMP-certified laboratory in accordance with Ph. Eur. It was expected that culture media containing preparation samples would not show microbial growth.

#### 2.4.10 Determination of Residual Ethanol Content

To keep the ethanol concentration in the final product lower than 10% v/v, the volume of physiological saline in the final formulation was increased to 8.6 mL, so that the use of 1.5 mL of 60% ethanol is inevitably less than 9% v/v. In addition, gas chromatography (GC) analyses on the 3 standardized validation batches were performed on a GC-2010 AF instrument (Shimadzu, Japan) to quantify final ethanol concentration.

### 2.5 Reproducibility of [^68^Ga]Ga-PSMA-11 Synthesis

After validation of the fully automated radiolabeling protocol for [^68^Ga]Ga-PSMA-11 preparation, the final activity concentration of all the preparations made over the 9 months of use of a single ^68^Ge/^68^Ga generator was recorded.

### 2.6 Transposition of the Automated Radiolabeling Procedure to the Preparation of [^68^Ga]Ga-PSMA-617

PSMA-617 was purchased from MedChem Express (NJ, USA). For [^68^Ga]Ga-PSMA-617 preparation, the automated sequence described above was used unchanged. The radiolabeling reaction involved 40 µg of vector. Among the reagents and consumables, the Sep-Pak^®^ C_18_ SPE cartridge (360 mg sorbent per cartridge) was replaced by a Sep-Pak^®^ lite C_18_ SPE cartridge (130 mg sorbent per cartridge). TLC and HPLC conditions for RCP determination of [^68^Ga]Ga-PSMA-617 were the same as for [^68^Ga]Ga-PSMA-11.

### 2.7 Transposition of the Automated Radiolabeling Procedure to the Preparation of [^68^Ga]Ga-PSMA-I&T

PSMA-I&T was purchased from ABX (Germany). HEPES was purchased from Merck (Germany) and was of the highest available purity grade. For [^68^Ga]Ga-PSMA-I&T preparation, the automated sequence described above was used unchanged. The radiolabeling reaction involved 40 µg of vector. Among the reagents and consumables, ammonium acetate 0.08 M was replaced by HEPES 2.7 M as the reaction buffer. TLC and HPLC analytical conditions for RCP determination of [^68^Ga]Ga-PSMA-I&T were the same as for [^68^Ga]Ga-PSMA-11. The residual HEPES content in the final preparation was determined according to the standard TLC method listed in Ph. Eur. monographs [35]. Briefly, two separate spots (40 µL) of a 49.5 µg/mL HEPES reference solution (*i.e*., 500 µg in 10.1 mL) and test solution (final [^68^Ga]Ga-PSMA-I&T) were deposited with capillary tubes on a TLC silica gel F254 plate coated with aluminum foil and dried during 10 min in a 37°C oven. The TLC was then developed in a chamber containing an acetonitrile/water/ methanol solution (75:15:10) as the mobile phase. The development was stopped when the front of the mobile phase was 0.5 cm below the top end of the plate. Finally, HEPES was visualized after treatment of the developed TLC plate by exposure to iodine vapor. A 200 cm^3^ hermetically sealed iodine chamber containing a mixture of 2 g of solid iodine and 8 g of silica was used; complete burying of the TLC plate under the iodine/silica mixture for 5 min at 37°C allowed optimal revelation. Any spot had to be less intense than the reference solution spot.

## 3 RESULTS AND DISCUSSION

### 3.1 Automated Radiolabeling Protocol

The fully automated production of ^68^Ga-labeled PSMA ligands on the GAIA^®^ module was completed in 27 min, from the start of the synthesis to the delivery of the radiolabeled compound in the product vial. The GMP-compliant sequence relied on two “bind and elute” approaches (for ^68^Ga eluate concentration and radiocomplex purification) separated by an 8-minute heating step to allow radiolabeling. Of note, the use of an SCX cartridge eliminates the influence of the volume of ^68^Ga eluate (and, therefore, the generator model), and could even allow eluates from more than one ^68^Ge/^68^Ga generator to be processed for the same synthesis [68-70]. Therefore, this automated protocol potentially displays the necessary qualities for straightforward adaptation to the 68Ga radiolabeling of other chelator-bearing agents, for which preparation would follow the steps outlined in Table **1**.

### 3.2 Radio-HPLC Method Validation

The analytical conditions described above allowed the identification of two main peaks on [^68^Ga]Ga-PSMA-11 radio-HPLC chromatograms attributable to the two major diastereomeric forms of the radiocomplex [24]. As the mean retention times of the two main peaks of the [^nat^Ga]Ga-PSMA-11 reference solution detected in UV were comparable to those obtained in radiochemical detection with [^68^Ga]Ga-PSMA-11 (8’00 *vs* 8’05 for isomer 1 and 8’26 *vs* 8’29 for isomer 2), the chemical species identity was confirmed. A difference of 3-5 seconds was observed between the UV and radio measurements as the detectors were connected in series.

**Fig. (3):**
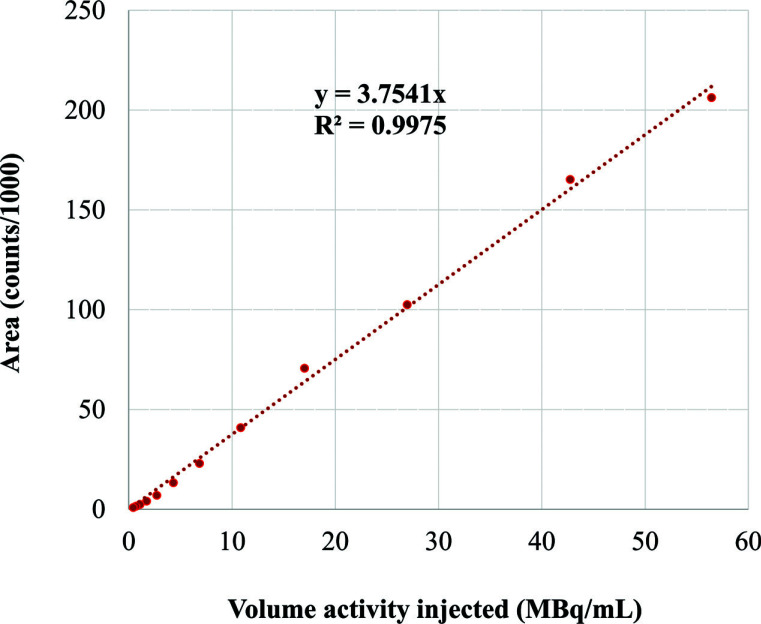
Concentration–peak area linear regression for RAD detector.

Results from the validation of the [^68^Ga]Ga-PSMA-11 radio-HPLC control method are summarized in Table **2**. Linearity of the radioactive detector was confirmed over the volume activity range 1-56 MBq/mL, with a good correlation coefficient (R^2^ = 0.9975) (Fig. **3**). Sufficient resolution (R > 3.31) was observed between isomer 1 of [^68^Ga]Ga-PSMA-11 and the nearest radio-impurity (^68^Ga radio-impurity 3), as well as between isomers 1 and 2 (R > 1.99), supporting the specificity of the method.

Successive RCP analyses of a [^68^Ga]Ga-PSMA-11 preparation showed very low variability in ^68^Ga radio-impurity and [^68^Ga]Ga-PSMA-11 isomers proportions (%CV from 0.069 to 0.19 and from 0.064 to 0.32, respectively), confirming that the method is repeatable. Slightly higher coefficients of variation were observed for the two ^68^Ga^3+^ peaks (%CV from 1.39 to 1.58), probably due to their very small surface area. Good recovery (>95%) of the injected activity at the column outlet confirmed the accuracy of the method. LOQ and LOD for isomer 2 were reached with [^68^Ga]Ga-PSMA-11 preparations of 8 MBq/mL and 1.05 MBq/mL, respectively. LOQ and LOD for the smallest ^68^Ga^3+^ peak were reached with solutions containing 228 kBq/mL and 97 kBq/mL of [^68^Ga]Ga^3+^, respectively. The total activity of the preparations used in these assays was systematically lower than the 150 MBq threshold set previously. The high-resolution test did not reveal any additional peaks when the solvent gradient time was doubled. Thus, HPLC discrimination of radioactive compounds contained in the final [^68^Ga]Ga-PSMA-11 solution was validated.

### 3.3 Production of [^68^Ga]Ga-PSMA-11 Validation Batches

Overall quality control specifications and results for the three [^68^Ga]Ga-PSMA-11 validation batches are presented in Table **3**. At the end of the synthesis, the final vial contained the mean activity of 660.7 ± 56.1 MBq, representing an average specific activity of 66.1 ± 5.6 MBq/µg of PSMA-11. These three validation batches were produced with a mean RCY of 68.3% ± 2.1%. Each final product appeared as a clear, colorless solution, with a pH of 6-7, as expected for a mixture of 0.9% saline and ethanol.

Gamma-spectrometry analysis performed on the validation batches identified the expected energy peaks, confirming radionuclide identity. Similarly, successive activity measurements on a sample from each validation batch found an average half-life of 69.16 ± 2.03 min (gamma counter linearity: R^2^ = 0.994), compatible with the radionuclide ^68^Ga. As expected from a radiopharmaceutical-grade ^68^Ga eluate, the final preparation displayed excellent radionuclidic purity, with ^68^Ge and γ-emitting impurities in a mean proportion of 2.06 × 10^-4^% ± 1.16 × 10^-4^%.

[^68^Ga]Ga-PSMA-11 validation batches were produced with high RCP, especially when measured by radio-TLC (mean RCP = 97.72% ± 0.04%). RCP values measured by radio-HPLC were slightly lower (from 91.24% to 94.81%), primarily due to the higher sensitivity of the method that detected 4 distinct radio-impurities, in addition to free ^68^Ga^3+^. General aspects of the chromatograms obtained when measuring [^68^Ga]Ga-PSMA-11 RCP are shown in Fig. (**4**).

**Fig. (5):**
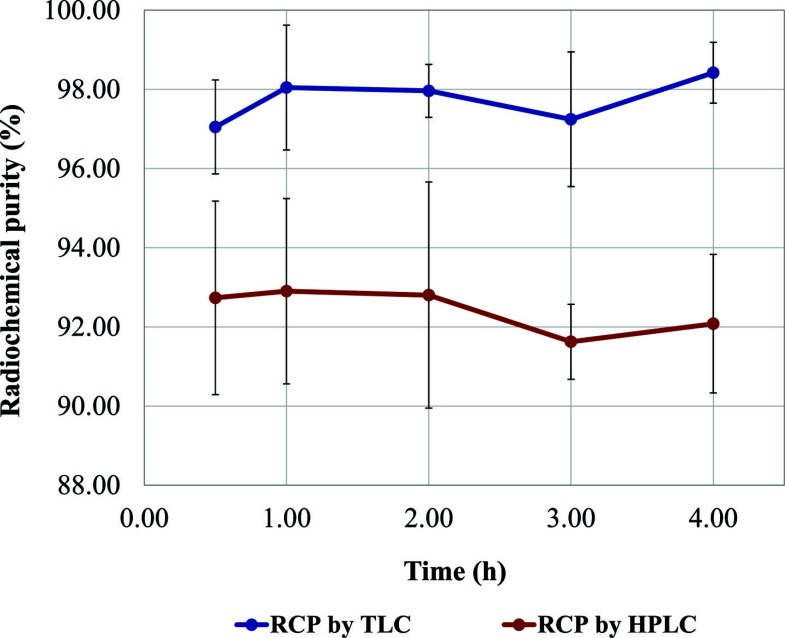
Time course of [^68^Ga]Ga-PSMA-11 RCP during stability tests, assessed by radio-TLC and radio-HPLC.

Radiochemical stability assessment on the final product, measured by radio-TLC and radio-HPLC, showed a steady mean RCP over a period of 4 h (Fig. **5**), proving the strength of the radiometal-chelator association. Indeed, this result was expected in view of the high stability constant of the Ga(III)-HBED complex (log Kd = 38.5 [71, 72]), which forms with rapid kinetics at room temperature.

Successive measurements of decay-corrected volume activities over 4 h were consistent, both within the same batch and between the three [^68^Ga]Ga-PSMA-11 validation batches (mean volume activity = 59.7 ± 3.2 MBq/mL). Coefficient of variation between measurements of a single sample were <5%, confirming the absence of significant radiocomplex adsorption on the vial surfaces.

Although not expected to exceed 9% of the total final volume, the average residual ethanol quantity in the three validation batches was measured by GC at 7.42% ± 0.23%, confirming the intended <10% value.

Regarding microbiological evaluation, the limulus amebocyte lysate test for bacterial endotoxin resulted in < 1 EU/mL in the three [^68^Ga]Ga-PSMA-11 validation batches. Similarly, sterility testing conducted on fluid thioglycollate medium and tryptic soy broth treated with samples of [^68^Ga]Ga-PSMA-11 and incubated at 30-35°C and 20-25°C, respectively, validated the sterility of the radiopharmaceuticals.

**Fig. (6):**
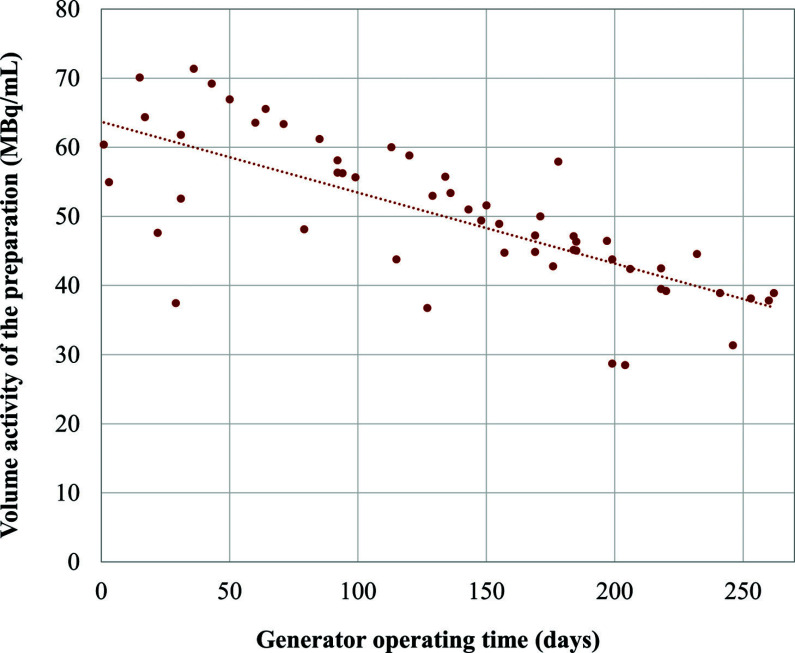
Activity concentration values of [^68^Ga]Ga-PSMA-11 preparations produced over the 9-months use of a single ^68^Ge/^68^Ga generator.

Following the approval of these batches, [^68^Ga]Ga-PSMA-11 was used for patients within our center. At the end of each synthesis, pH, RCP by TLC and HPLC, and bacterial endotoxins were measured as release QCs prior to patient injection. Over the 9-months use of a single ^68^Ge/^68^Ga generator, the average volume activity of the [^68^Ga]Ga-PSMA-11 preparations produced ranged between 70 and 40 MBq/mL (55 syntheses), with satisfactory reproducibility (Fig. **6**). However, in some syntheses, specific steps such as purifications could lead to a significant loss of activity.

### 3.4 Application of the Radiolabeling Method to the Preparation of [^68^Ga]Ga-PSMA-617

**Fig. (7):**
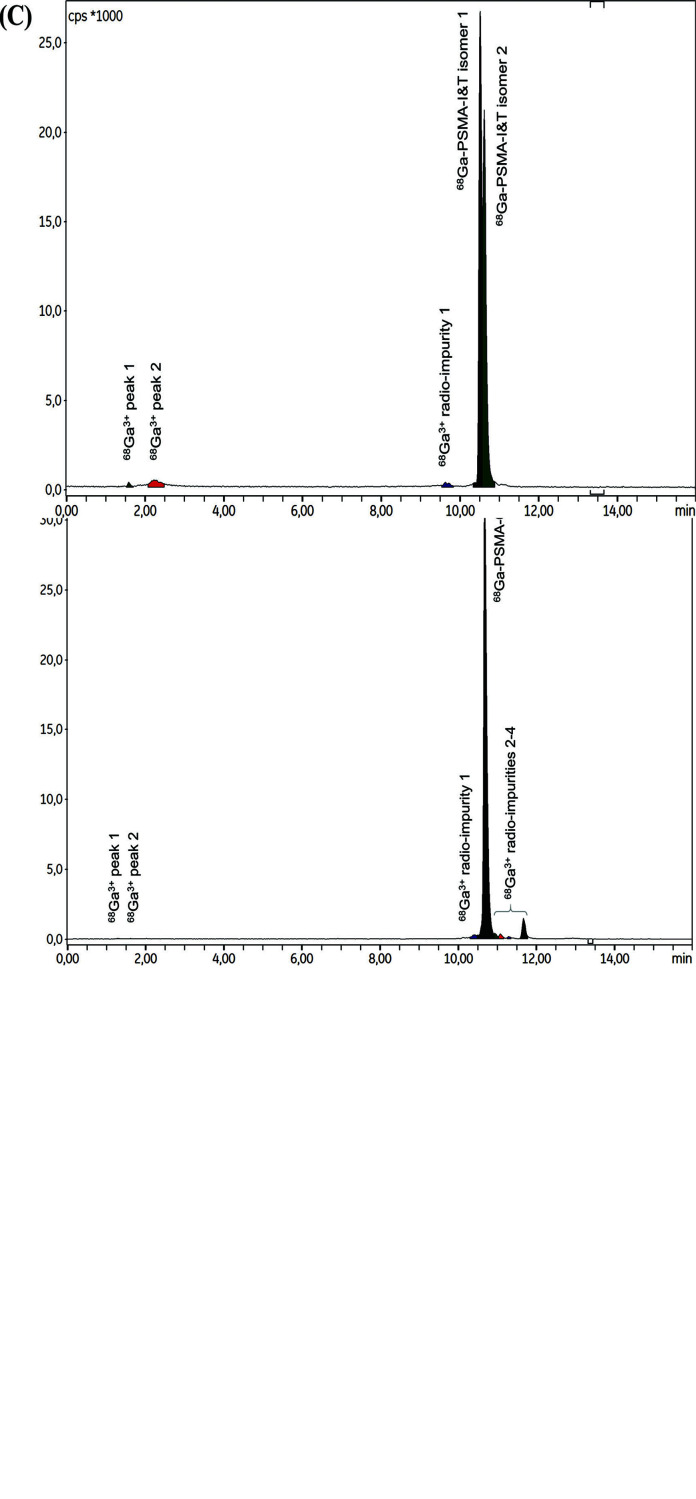
Representative radio-HPLC chromatograms for [^68^Ga]Ga-PSMA-11 (**A**), [^68^Ga]Ga-PSMA-617 (**B**) and [^68^Ga]Ga-PSMA-I&T (**C**).

The ^68^Ga radiolabeling protocols for PSMA-617 reported in the literature mention the use of different vector amounts [38, 72]. Initial experiments with the automated method described above involving only 20 µg of PSMA-617 were conducted, and were associated with low RCY (<20%). This was due to both the retention of a significant fraction of radioactivity on the C_18_ cartridge and the elimination of a substantial amount of free ^68^Ga^3+^ in the waste vial. In addition, the radiolabeled product was associated with a poor RCP (<80%). The use of 40 µg PSMA-617 solved this issue (mean RCP by TLC = 97.4% ± 1.1%; mean RCP by HPLC = 93.6% ± 0.9%, n = 3), this amount remaining lower than the maximum PSMA-617 vector quantities reported in the literature (*i.e*., 50 µg [72]). At the final purification step by SPE, the use of a Sep-Pak^®^ C_18_ cartridge containing 360 mg sorbent was associated with late elution of [^68^Ga]Ga-PSMA-617 after all 60%ethanol (~1.5 mL) had passed through the cartridge. Comparatively, [^68^Ga]Ga-PSMA-11 was fully eluted after the passage of only two-thirds of the ethanol volume (~1 mL). The use of a smaller cartridge containing only 130 mg sorbent was a convenient solution for both retaining the radiolabeling product after the reaction and eluting it more effectively after a smaller proportion of the total ethanol eluent passed through the cartridge (Table **4**). Of note, this change required a closer control of the formulation step completion, as a smaller cartridge implied greater resistance to liquid flow and, therefore, an increased throughput time for the 8.6 mL saline. The radio-TLC analysis of [^68^Ga]Ga-PSMA-617 showed no difference when compared to [^68^Ga]Ga-PSMA-11. In radio-HPLC analysis, the retention time of [^68^Ga]Ga-PSMA-617 was significantly longer compared to the two [^68^Ga]Ga-PSMA-11 isomers (10.68 min and 8.30/8.62 min, respectively) (Fig. **7**). This may be due to the less hydrophilic properties of PSMA-617 compared with PSMA-11 (logP = -3.4 for [^68^Ga]Ga-PSMA-617 and logP = -4.3 for [^68^Ga]Ga-PSMA-11 [73]). It can be assumed that this difference in physicochemical properties is partly due to the presence of a naphthyl moiety in the structure of PSMA-617, which slightly increases lipophilicity.

### 3.5 Application of the Radiolabeling Method to the Preparation of [^68^Ga]Ga-PSMA-I&T

For the ^68^Ga radiolabeling of PSMA-I&T, the strict transposition of the conditions retained for PSMA-11 initially resulted in the retention of ~1/3 of the post-reaction activity on the C_18_ cartridge, which could not be eluted by the 1.5 mL of ethanol 60%. The remaining ~2/3 of the activity was directly eliminated in the waste vial without retention on the SPE cartridge. Replacing the SPE cartridge with a C_18_ 130 mg or a less lipophilic OASIS HLB 130 mg cartridge did not solve this issue. The pH of the reaction medium was checked and measured at 3.2, demonstrating good compatibility with ^68^Ga radiolabeling. Most of the [^68^Ga]Ga-PSMA-I&T preparation conditions reported in the literature mention the use of high-concentration HEPES buffer (2.7 M) for radiolabeling [27, 28]. Thus, ammonium acetate 0.08 M buffer was replaced in the original PSMA-11 radiolabeling protocol with HEPES 2.7 M buffer, allowing efficient transposition to PSMA-I&T (Table **4**). As expected, the results obtained with this buffer were considerably better, with a 65% RCY and a >95% RCP in HPLC.

Notably, the radio-HPLC spectrum of [^68^Ga]Ga-PSMA-I&T was characterized by a particular profile (Fig. **7C**), already described in the literature [74] and probably due to the formation of [^68^Ga]Ga-(R)- and (S)-DOTAGA isomers (mean retention times: 10.51/10.61 min, respectively) [75]. The final SPE purification step should allow the elimination of the HEPES buffer involved in the radiolabeling reaction. To ensure that HEPES traces in the final preparation remained below the thresholds recommended by the Ph. Eur. (< 500 µg/injected dose, *i.e*., 10.1 mL if the total preparation volume was injected into a single patient), the semi-quantitative TLC control method recommended by Ph. Eur. [63] was used, and confirmed a concentration below the specified limit (see Supplementary materials). A 10-fold lower molarity (0.3 M) of HEPES was tested to investigate the influence of the buffer concentration on the radiolabeling outcome, but only ~30% of the radioactivity involved in the reaction was effectively recovered as [^68^Ga]Ga-PSMA-I&T at the end of the synthesis.

This underlines the importance of the nature and concentration of the buffer solution in the reliability of the radiolabeling process. Therefore, although the general automated radiolabeling protocol developed here can be transposed to the various PSMA ligands studied, a detailed evaluation of the influence of each reaction parameter involved in ^68^Ga radiolabeling seems worthwhile to be performed for every specific vector molecule in order to identify the most suitable preparation conditions. In addition, the application of this automated radiolabeling protocol to the synthesis of [^68^Ga]Ga-PSMA-617 or [^68^Ga]Ga-PSMA-I&T for human use would require the use of pharmaceutical-grade reagents and ingredients, such as those complying with Ph. Eur. specifications.

## CONCLUSION

In the present work, a GMP-compliant synthesis method of [^68^Ga]Ga-PSMA-11 on the GAIA^®^ module was validated, as well as the associated QCs. This protocol was successfully applied to the related prostate-targeting molecules PSMA-617 and PSMA-I&T to prepare the corresponding ^68^Ga complexes, with slight adaptations of the reagents and disposables used. This highlights that although all PSMA ligands are directed at the same target, each of these molecules offers specific physicochemical characteristics that should be considered when developing an automated ^68^Ga radiolabeling method. Furthermore, the variety of PSMA ligands tends to continuously expand, given the ongoing interest in these compounds as molecular targeting agents in nuclear medicine. In this context, new vectors susceptible to automated radiolabeling continue to be developed and evaluated in humans, such as PSMA-11-derived [^68^Ga]Ga-P16-093 [40, 76-80], NOTA derivative [^68^Ga]Ga-P137 [81, 82] or the latest FDA-approved radiohybrid agent flotufolastat [83, 84].

## Figures and Tables

**Fig. (4):**
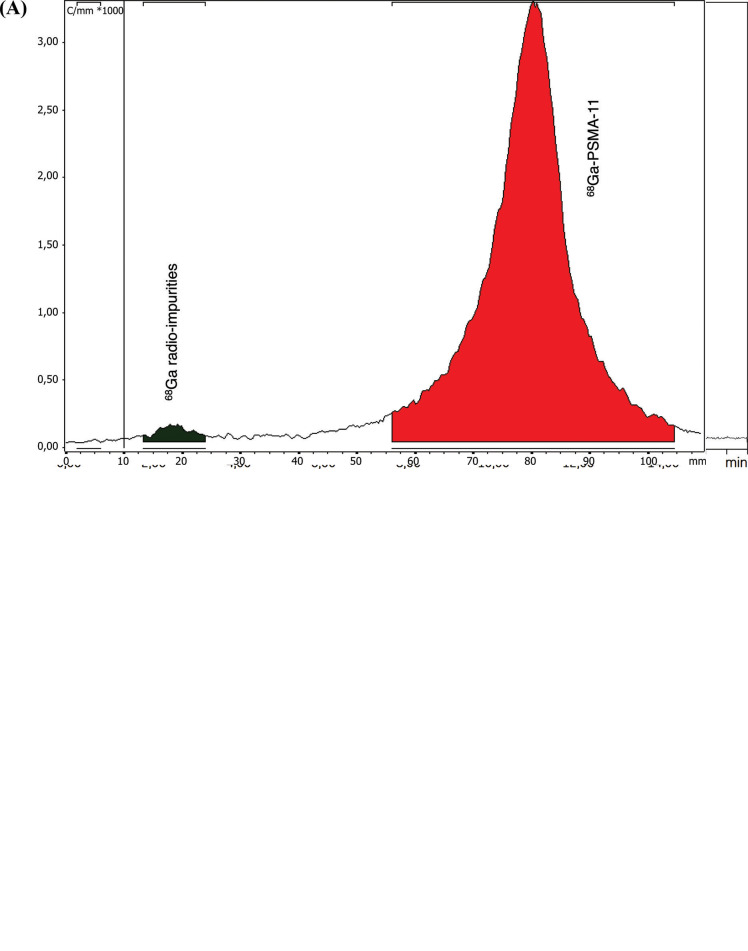
Representative radio-TLC (**A**) and radio-HPLC (**B**) chromatograms for [^68^Ga]Ga-PSMA-11.

**Table 1:** Summary of process steps of the automated synthesis of [^68^Ga]Ga-PSMA-11.

1	Drying of the tubing set and of the C_18_ SPE cartridge with filtered air.
2	Kit integrity test (pressurization >1500 mbar and pressure reduction of not more than 400 mbar over 20 sec).
3	Addition of PSMA-11 solubilized in the buffer to the reaction vial.
4	Conditioning of SCX and C_18_ cartridges with WFI and purging of the tubing with filtered air.
5	Generator elution: vacuum building-up and natural elution for 60 sec followed by final active elution for 140 sec.
6	The passing of the eluate through the SCX cartridge and retention of [^68^Ga]Ga^3+^ on the cartridge.
7	Washing of the SCX cartridge with WFI, purge with filtered air and elution of [^68^Ga]Ga^3+^ with 0.5 mL NaCl 5 M pH 1 to the reaction vial.
8	Labeling at 97°C during 8 min.
9	Transfer of the reaction mixture from the reaction vial onto the C_18_ SPE cartridge.
10	Washing of the reaction vial with 10 mL WFI and transfer from the reaction vial onto the C_18_ SPE cartridge.
11	C_18_ cartridge rinsing with WFI and purging of the tubing with filtered air.
12	[^68^Ga]Ga-PSMA-11 elution to the product vial with alternating ethanol 60% (total 1.5 mL) and NaCl 0.9% (total 1.5 mL).
13	Formulation of the final product with 7.1 mL NaCl 0.9%.
13	Product vial release and filter integrity testing.
14	Closing all valves, end of synthesis.

**Table 2:** Summary of the specifications and results for the validation of the radio-HPLC method (RAD detection).

**Parameter**	**Criteria**	**Specifications**	**Results**
Identity	RRT	0.95–1.05	Isomer 1:1.01 Isomer 2:1.006
Linearity	R^2^ value	R^2^ ≥ 0.99	0.9975
Specificity	R value	R > 1.5	1.99–4.76
Repeatability (%)	RRT	CV ≤ 2	< 1.8
Accuracy (%)	Recovery	≥ 95	95.6 ± 0.6
LOQ (MBq/mL)	Radio detection	Signal/noise ratio > 10	Isomer 2:8 ^68^Ga^3+^:0.228
LOD (MBq/mL)	Radio detection	Signal/noise ratio > 3	Isomer 2:1.05 ^68^Ga^3+^:0.097
HR test	Radio detection	No extra signal	No extra signal

**Table 3:** Summary of the product specifications of the three [^68^Ga]Ga-PSMA-11 validation batches.

**Test**	**Specifications**	**Batch 1**	**Batch 2**	**Batch 3**
**Appearance**	Clear, colorless solution	Clear, colorless solution	Clear, colorless solution	Clear, colorless solution
**Identification**				
Energy of gamma photons (MeV)	0.511 and 1.077	0.511 and 1.077	0.511 and 1.077	0.511 and 1.077
Half-life (min)	61 – 75	71.23	67.33	68.09
Chemical identity	RRT between 0.95 and 1.05	Isomer 1: 1.010 Isomer 2: 1.006	Isomer 1: 1.010 Isomer 2: 1.006	Isomer 1: 1.008 Isomer 2: 1.006
**pH**	4–8	6	7	6
**Sterility**	Sterile	Sterile	Sterile	Sterile
**Bacterial endotoxins**	< 12.5 EU/mL	< 1 EU/mL	< 1 EU/mL	< 1 EU/mL
**Radionuclidic Purity**				
(^68^Ga) Gallium	≥99.9%	99.999669%	99.999814%	99.999898%
(^68^Ge) Germanium and γ-emitting impurities	≤0.001%	0.000331%	0.000186%	0.000102%
**Radiochemical Purity**				
[^68^Ga]Ga-PSMA-11 (HPLC)	≥91%	94.81%	91.24%	91.24%
[^68^Ga]Ga^3+^ (HPLC)	≤2%	1.47%	1.05%	1.20%
[^68^Ga]Ga-PSMA-11 (TLC)	≥95%	97.69%	97.71%	97.76%
[^68^Ga]gallium impurities (TLC)	≤5%	2.31%	2.29%	2.24%
**Filter integrity test**	Bubble point measurement >2500 mbar	3759 mbar	4107 mbar	3963 mbar
**Radioactivity**	>150 MBq	721 MBq	610 MBq	651 MBq
**Specific activity**	-	72.1 MBq/µg	61.0 MBq/µg	65.1 MBq/µg
**Radiochemical yield**	-	70%	66%	69%
**Stability over 4 h**	-	≥92.71% (HPLC) ≥95.69% (TLC)	≥91.0% (HPLC) ≥95.28% (TLC)	≥91.09 (HPLC) ≥97.75% (TLC)
**Adsorption in the product vial (%CV)**	-	4.73	3.11	4.72
**Ethanol content**	<10%	7.15%	7.55%	7.55%

**Table 4:** Summary of changes made to the ^68^Ga radiolabeling protocol of PSMA-11 for transposition to PSMA-617 and PSMA-I&T.

**Parameter**	**PSMA-11**	**PSMA-617**	**PSMA-I&T**
Vector amount	10 µg	40 µg	40 µg
Reaction buffer	Ammonium acetate 0.08 M	Ammonium acetate 0.08 M	HEPES 2.7 M
SPE cartridge	Sep-Pak^®^ C_18_ 360 mg sorbent	Sep-Pak^®^ lite C_18_ 130 mg sorbent	Sep-Pak^®^ C_18_ 360 mg sorbent

## Data Availability

The data that support the findings of this study will be made available from the corresponding author [C.F.] upon request.

## LIST OF ABBREVIATIONS

CPM: Counts Per Minute
GMP: Good Manufacturing Process
HPLC: High-Performance Liquid Chromatography
LOD: Limit of Detection
LOQ: Limit of Quantification
PSMA: Prostate-specific Membrane Antigen
RCP: Radiochemical Purity
RCY: Radiochemical Yield
TLC: Thin-Layer Chromatography
