# Supplementary material for: “One Method to Label Them All”: A Single Fully Automated Protocol for GMP-Compliant 68Ga Radiolabeling of PSMA-11, Transposable to PSMA-I&T and PSMA-617
Source: Curr Radiopharm. 2024 Feb 28;17(3):285–301. doi: 10.2174/0118744710293461240219111852 (PMC11348474; doi:10.2174/0118744710293461240219111852)
Supplement: Supplementary file 1 — Supplementary material is available on the publisher’s website along with the published article. [file CRP-17-285_SD1.pdf]

SUPPLEMENTARY MATERIAL

"One Method to Label Them All": A Single Fully Automated Protocol for GMP-Compliant ^{68}Ga Radiolabeling of PSMA-11, Transposable to PSMA-I&T and PSMA-617

Juliette Fouillet[1], Charlotte Donzé[1], Emmanuel Deshayes[1,2], Lore Santoro[1,2], Léa Rubira[1] and Cyril Fersing[1,3,*]

[1]Nuclear Medicine Department, Institut Régional du Cancer de Montpellier (ICM), Univ. Montpellier, Montpellier, France; [2]Institut de Recherche en Cancérologie de Montpellier (IRCM), INSERM U1194, Univ. Montpellier, Institut Régional du Cancer de Montpellier (ICM), Montpellier, France; [3]IBMM, Univ. Montpellier, CNRS, ENSCM, Montpellier, France

1. Detailed automated synthesis sequence for [^{68}Ga]Ga-PSMA-11 radiolabeling

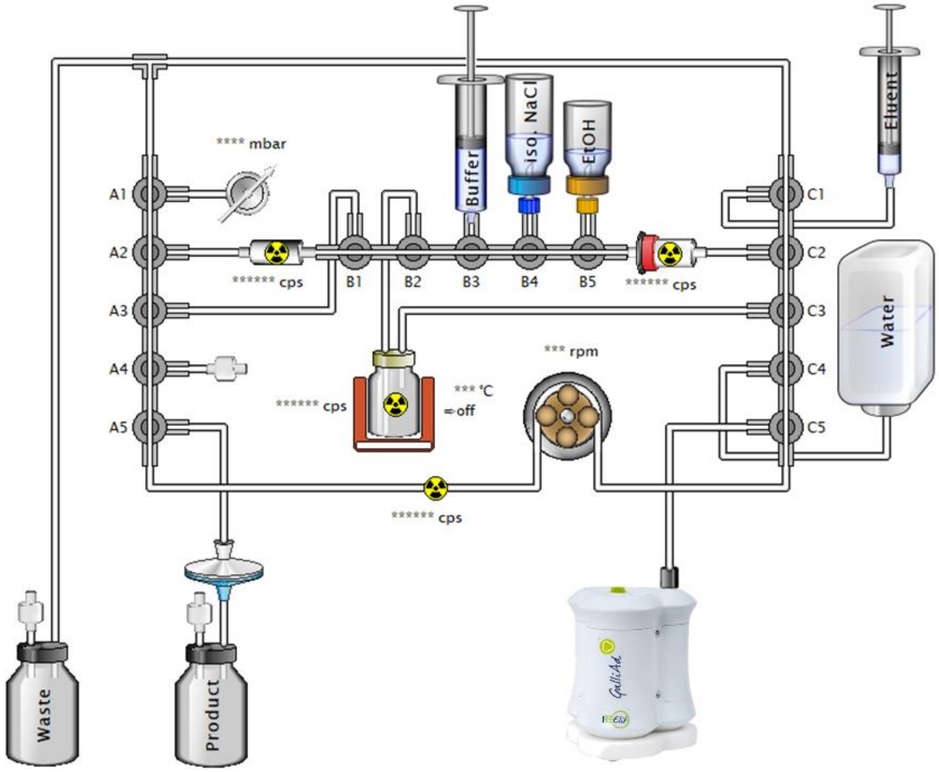

	A1	A2	A3	A4	A5	B1	B2	B3	B4	B5	C1	C2	C3	C4	C5	Pump	Heater	Wait Condition	Wait Timeout	Measurement	Description (English)
0	0	0	0	0	0	0	0	0	0	0	0	0	0	0	0	off	off				Initial State
1	0	0	3	1	0	1	0	0	0	0	3	3	0	0	0	250	off	Fixed delay	15		C18 Purge
2	1	2	2	1	0	2	0	0	0	0	0	3	2	0	0	250	off	Pressure > 1500mbar	180		Kit Integrity Test: Pressurizing
3	1	2	2	0	0	2	0	0	0	0	0	3	3	0	0	off	off	Fixed delay	5		Kit Integrity Test: Equilibration
4	1	2	2	0	0	2	0	0	0	0	0	3	3	0	0	off	off	Fixed delay	15	Gaia–Pressure changes less than 400.0mbar	Kit Integrity Test: Measuring
5	2	2	2	2	0	2	0	0	0	0	0	2	2	0	0	off	off	Fixed delay	5		Kit Integrity Test: Venting
6	0	0	0	1	0	0	0	0	0	0	3	0	0	0	0	100	off	Fixed delay	3		NaCl Syringe Preparation
7	0	0	0	1	0	0	1	3	0	0	0	0	3	0	0	200	off	Fixed delay	4		Buffer Addition: Preparation
8	0	0	0	1	0	0	1	3	0	0	0	0	1	0	0	off	off	Fixed delay	2		Buffer Addition: Preparation Venting
9	0	0	0	1	0	0	1	3	0	0	0	0	3	0	0	-250	off	Fixed delay	25		Buffer addition
10	0	0	0	2	0	0	0	0	0	0	0	0	3	0	0	off	off	Fixed delay	3		Venting
11	1	1	0	0	0	0	0	0	0	0	0	1	0	3	0	-150	off	Fixed delay	30		Cartridge Activation: SCX + C18
12	1	1	1	0	0	1	0	0	0	0	0	1	0	3	0	-150	off	Fixed delay	10		Cartridge Activation: C18
13	1	1	3	0	0	1	0	0	0	0	0	0	0	0	0	250	off	Fixed delay	5		Cartridge Activation: Purge Preparation
14	1	0	3	1	0	1	0	0	0	0	0	0	0	0	0	250	off	Fixed delay	5		Cartridge Activation: Purge 1
15	0	0	3	1	0	1	0	0	0	0	3	3	0	0	0	250	off	Fixed delay	10		Cartridge Activation: Purge 2
16	0	3	3	1	0	0	0	0	0	0	3	3	0	0	0	250	off	Fixed delay	10		Cartridge Activation: Purge 3
17	0	0	3	1	0	1	0	1	0	0	3	3	0	0	0	200	off	Fixed delay	15		pressurisation buffer syringe
18	0	0	3	1	0	1	0	0	0	0	3	3	0	0	0	off	off	Fixed delay	3		Cartridge Activation: Venting
19	0	0	0	0	0	0	0	0	0	0	3	3	0	0	0	off	off	User prompt	1200		Generator elution
20	0	3	1	0	0	3	0	0	0	0	3	3	1	0	1	-250	60	Fixed delay	7		Pre vacuum
21	0	3	1	0	0	3	0	0	0	0	3	3	1	0	3	-100	60	Fixed delay	40		Elution 60s trigger
22	0	3	1	0	0	3	0	0	0	0	3	3	1	0	3	off	60	Fixed delay	20		Natural elution
23	0	3	1	0	0	3	0	0	0	0	3	3	1	0	3	-100	60	Fixed delay	140		Finish elution
24	0	3	1	0	0	3	1	3	0	0	3	3	3	0	0	-200	60	Fixed delay	20		Wash line
25	0	0	0	0	0	3	1	3	0	0	3	3	3	0	0	off	60	Fixed delay	2		Equilibration
26	0	0	0	0	0	0	0	0	0	0	3	3	0	3	0	-100	60	Fixed delay	10		SCX Washing: Line
27	0	3	1	0	0	3	0	0	0	0	3	3	0	3	0	-100	60	Fixed delay	10		SCX Washing: Cartride
28	0	0	3	0	0	1	0	0	0	0	3	3	0	0	0	250	60	Fixed delay	5		SCX Washing: Line Purge Preparation
29	0	0	3	1	0	1	0	0	0	0	3	3	0	0	0	250	60	Fixed delay	15		SCX Washing: Line Purge
30	0	3	0	1	0	0	0	0	0	0	3	3	0	0	0	250	60	Fixed delay	10		SCX Washing: Cartridge Purge
31	0	0	0	0	0	0	0	0	0	0	3	0	0	0	0	-70	95	Fixed delay	12	Gaia–Manifold Detector 1	SCX Elution: Preparation 7 / Measuring SCX Activity
32	0	1	0	0	0	0	3	0	0	0	3	0	0	0	0	-20	95	Fixed delay	20		SCX Elution 1
33	1	1	0	0	0	0	3	0	0	0	3	0	3	0	0	-20	120	Fixed delay	60		SCX Elution 2
34	1	1	0	0	0	0	3	0	0	0	3	0	3	0	0	-250	97	Fixed delay	10	Gaia–Manifold Detector 1	SCX Elution: Purge / Measuring SCX post-elution
35	1	1	0	0	0	0	0	0	0	0	3	0	0	0	0	off	97	Fixed delay	10	Gaia–Reactor Detector	Labeling / Measuring Reactor Activity
36	1	1	0	0	0	0	0	0	0	0	3	0	0	0	0	off	97	Fixed delay	120		Labeling: Part 1
37	1	1	0	0	0	0	3	0	0	0	3	0	3	0	0	-40	97	Fixed delay	10		Labeling: Lift Line Purge
38	1	1	0	0	0	0	0	0	0	0	3	0	0	0	0	off	97	Fixed delay	340		Labeling: Part 2
39	1	1	1	0	0	1	3	0	0	0	0	0	1	3	0	-160	40	Fixed delay	12		Delution
40	1	1	1	0	0	1	1	0	0	0	0	1	3	0	0	250	40	Fixed delay	10		C18 Peptide Trapping: Preparation
41	1	1	1	1	0	1	1	0	0	0	0	1	3	0	0	100	40	Fixed delay	75		C18 Peptide Trapping
42	1	1	1	0	0	1	3	0	0	0	0	0	1	3	0	-250	40	Fixed delay	18		Rinsing Reaktor
43	1	1	1	0	0	1	1	0	0	0	0	1	3	0	0	250	40	Fixed delay	10		C18 Rinsing: Preparation
44	1	1	3	1	0	1	1	0	0	0	0	1	3	0	0	200	40	Fixed delay	60		C18 Rinsing
45	0	0	3	1	0	1	0	0	0	0	0	3	0	0	0	250	off	Fixed delay	5	Gaia–Reactor Detector	C18 Purge / Measuring Empty Reactor Activity
46	0	0	3	1	0	1	0	0	0	0	0	3	0	0	0	250	off	Fixed delay	5	Gaia–Manifold Detector 2	C18 Purge / Measuring C18 Activity
47	1	0	0	3	1	1	0	0	1	1	0	3	0	0	0	-15	off	Fixed delay	20		C18 Elution: EtOH
48	1	0	0	3	1	1	0	0	1	0	0	3	0	0	0	-15	off	Fixed delay	10		C18: Elution: Water
49	1	0	0	3	1	1	0	0	1	1	0	3	0	0	0	-15	off	Fixed delay	15		C18: Elution: EtOH
50	1	0	0	3	1	1	0	0	1	0	0	3	0	0	0	-15	off	Fixed delay	5		C18: Elution: Water
51	1	0	0	3	1	1	0	0	1	1	0	3	0	0	0	-15	off	Fixed delay	15		C18: Elution: EtOH
52	1	0	0	3	1	1	0	0	1	0	0	3	0	0	0	-15	off	Fixed delay	5		C18: Elution: Water
53	1	0	0	3	1	1	0	0	1	1	0	3	0	0	0	-15	off	Fixed delay	20		C18: Elution: EtOH
54	1	0	0	3	1	1	0	0	1	0	0	3	0	0	0	-150	off	Fixed delay	90		Formulation
55	1	0	1	3	1	1	0	0	0	0	0	3	0	0	0	-200	off	Fixed delay	20	Gaia–Manifold Detector 2	Formulation: Purge / Measuring C18 Activity Post_Elution
56	1	0	1	0	1	1	0	0	0	0	0	3	0	0	0	off	off	Fixed delay	5		Venting Preparation
57	1	0	2	0	0	1	0	0	0	0	0	2	0	0	0	off	off	User prompt	1		Filter Integrity Test: Preparation
58	0	0	0	0	1	0	0	0	0	0	0	0	0	3	0	-250	off	Fixed delay	30		Filter Integrity Test: Filter Purge
59	1	0	0	0	0	0	0	0	0	0	0	0	0	0	0	250	off	Fixed delay	5		Filter Integrity Test: Line Purge Preparation
60	1	0	0	1	0	0	0	0	0	0	0	0	0	0	0	250	off	Fixed delay	15		Filter Integrity Test: Line Purge 1
61	0	0	3	1	0	1	0	0	0	0	0	3	0	0	0	250	off	Fixed delay	25		Filter Integrity Test: Line Purge 2
62	0	0	3	0	0	1	0	0	0	0	0	3	0	0	0	-250	off	Fixed delay	15		Filter Integrity Test: Line Purge 3
63	1	0	0	0	2	0	0	0	0	0	0	0	0	0	0	-250	off	Pressure > 2500mbar	120		Filter Integrity Test: Pressurizing
64	1	0	0	0	2	0	0	0	0	0	0	0	0	0	0	-150	off	Pressure > 2900mbar	200		Filter Integrity Test: Approaching Bubble Point Value
65	1	0	0	0	2	0	0	0	0	0	0	0	0	0	0	-120	off	Sterile Filter Test	600		Filter Integrity Test: Measuring Bubble Point Value
66	2	0	0	2	2	0	0	0	0	0	0	2	2	0	0	off	off	Fixed delay	5		Venting
67	0	0	0	0	0	0	0	0	0	0	0	0	0	0	0	off	off	Stop Synthesis	5		Stop

2. Quality controls for [^{68}Ga]Ga-PSMA-11

2.1. Radionuclide identity in the validation batches

2.1.1. Gamma counter linearity

Volume activity (Bq/mL)	Delta T for decay correction (min)	^{68}Ga CPM	^{68}Ga corrected CPM
860000.000	0	3 451 899	3 451 899
430000.000	1	2 957 948	2 988 389
215000.000	2	1 675 854	1 710 524
107500.000	3	862 440	889 341
53750.000	4	450 345	469 171
26875.000	5	224 787	236 594
13437.500	6	111 899	118 989
6718.750	7	55 550	59 677
3359.375	8	27 413	29 753
1679.688	9	13 670	14 990
839.844	211	792	6 870
419.922	212	436	3 821
209.961	213	242	2 143
104.980	214	144	1 288
52.490	215	64	578
26.245	216	38	347
13.123	217	10	92

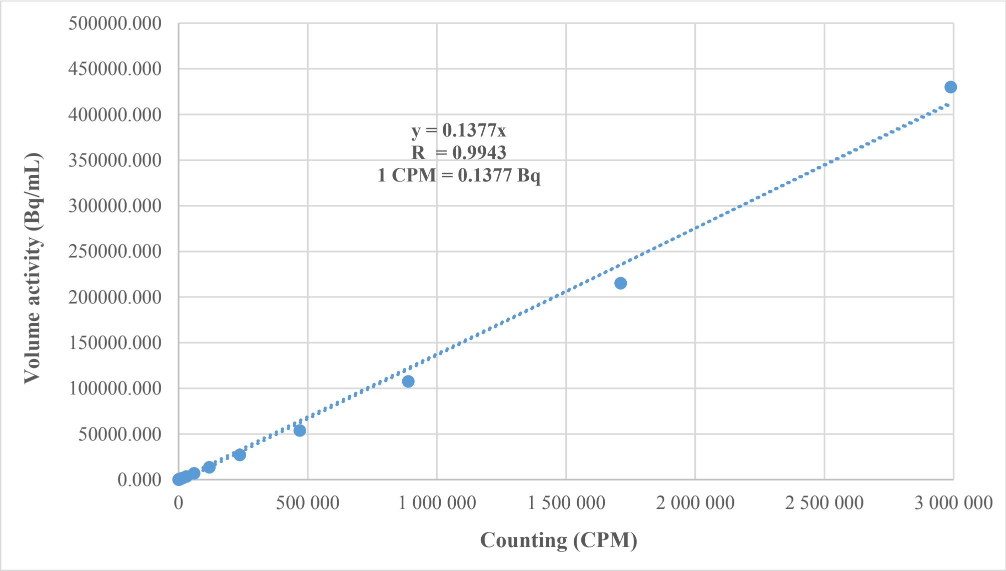

2.1.2. Gamma-spectrometry analyses of the [^{68}Ga]Ga-PSMA-11 validation batches

Batch 1:

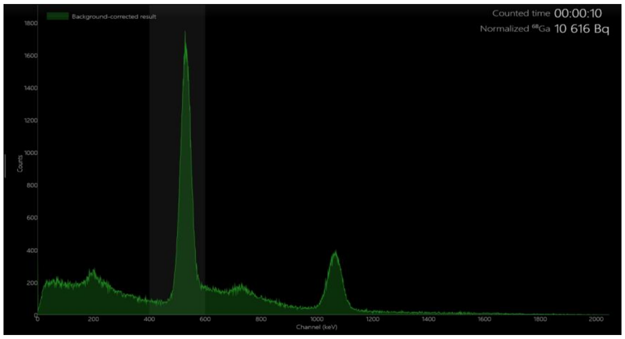

Batch 2:

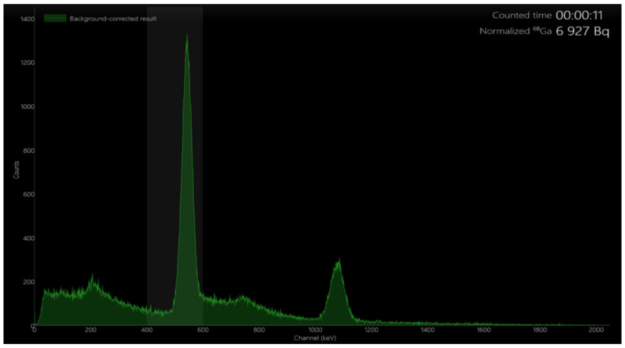

Batch 3:

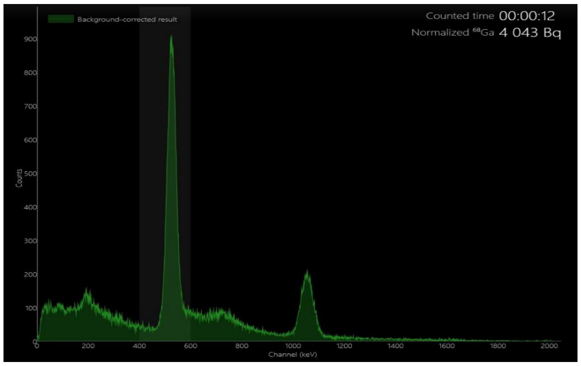

2.1.3. Half-life determination

Batch 1:

Time (min)	CPM	Calculated decay constant (min^{-1})	Calculated half-life (min)
0	166885	-	-
27	128540	0.009668875	71.69
53	101235	0.009431356	73.49
66	87872	0.009718467	71.32
85	73348	0.009671634	71.67
96	64633	0.009880969	70.15
107	57952	0.009884915	70.12
136	43573	0.009874006	70.20
			**Mean: 71.23 ± 1.22**

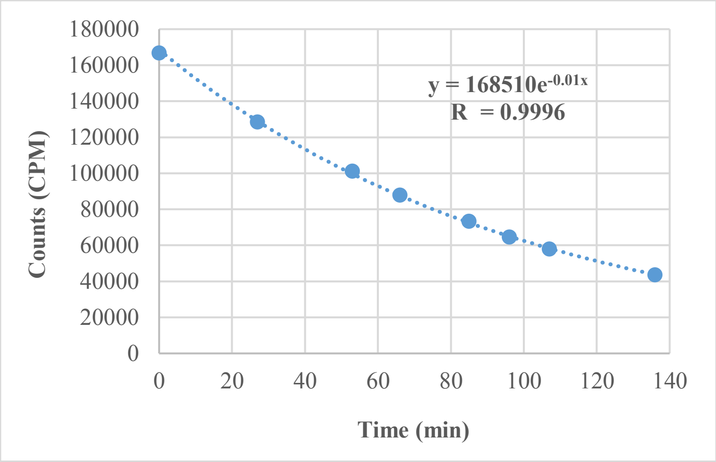

Batch 2:

Time (min)	CPM	Calculated decay constant (min-1)	Calculated half-life (min)
0	323820	-	-
11	288308	0.01055986	65.64
67	163399	0.01020887	67.90
116	98039	0.01030017	67.29
180	51561	0.01020786	67.90
254	24256	0.01020291	67.94
			**Mean: 67.33 ± 0.98**

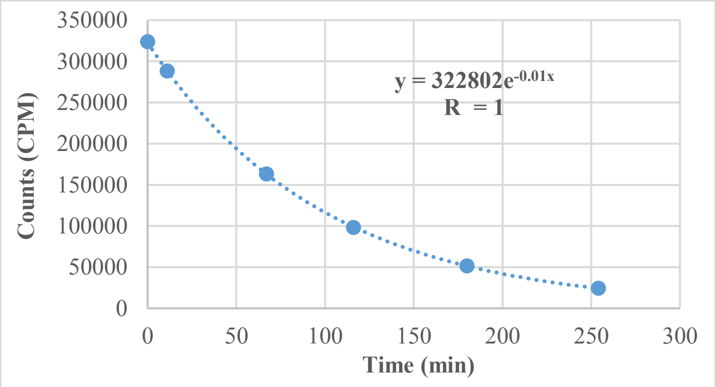

Batch 3:

Time (min)	CPM	Calculated decay constant (min^{-1})	Calculated half-life (min)
0	357 081	-	-
41	236 020	0.01009862	68.64
54	206 245	0.01016471	68.19
67	180 828	0.01015546	68.25
124	100 363	0.01023522	67.72
252	27 015	0.01024429	67.66
			**Mean: 68.09 ± 0.40**

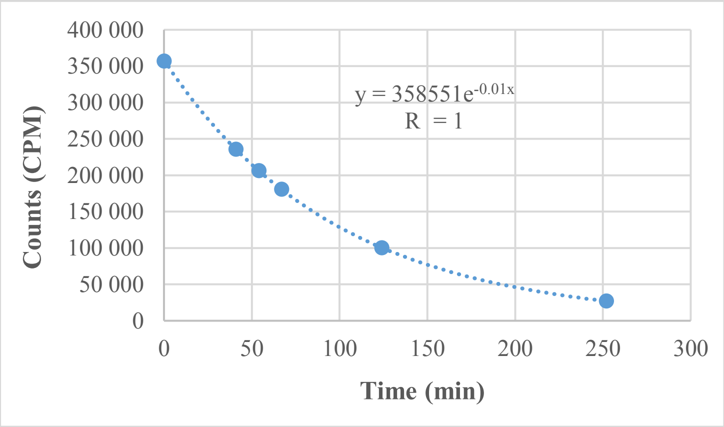

Overall mean of half-lives calculated for the 3 batches: **69.16 ± 2.03 min**

2.2. Radionuclide purity of the validation batches

	Sample activity at t_0 (MBq)	Decay time (min)	Gamma counter measurement after decay (CPM)	Conversion to activity (Bq); 1 CPM = 0.1377 Bq	Calculated radionuclide purity (%)
**Batch 1**	2.55	2895	61	8.40	99.9996690
**Batch 2**	3.87	2679	52	7.16	99.9998141
**Batch 3**	5.18	2810	38	5.23	99.9998985

2.3. Radio-HPLC method validation

2.3.1. RAD detection linearity

Volume activity (MBq/mL)	Area under curve (counts/1000)
56.42	206.275
42.74	165.256
26.96	102.5297
17.00	70.61274
10.84	40.9334
6.83	22.968
4.31	13.3074
2.72	7.029115
1.71	4.12895
1.09	2.45339
0.69	1.56849
0.43	0.88494

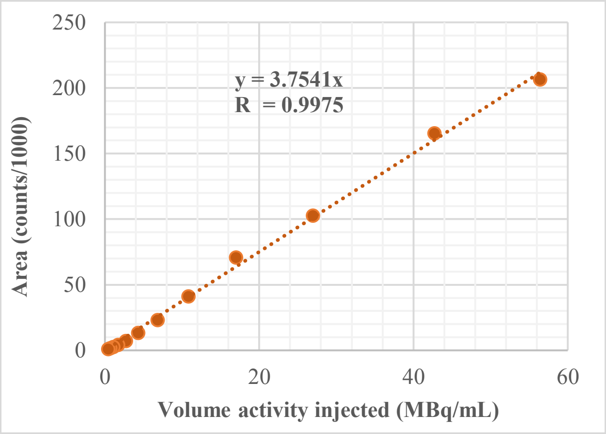

2.3.2. Specificity

Injection time	Retention time (min)								Resolution	
	$^{68}Ga^{3+}$ peak 1	$^{68}Ga^{3+}$ peak 2	^{68}Ga-impurity 1	^{68}Ga-impurity 2	^{68}Ga-impurity 3	^{68}Ga-impurity 4	^{68}Ga-PSMA isomer 1	^{68}Ga-PSMA isomer 2	Impurity 3 vs Isomer 1	Isomer 1 vs Isomer 2
Batch 1										
t_0	1.16	1.39	5.35	6.54	7.34	9.21	8.05	8.32	**3.79**	**2.15**
t_1	1.16	1.40	5.35	6.55	7.34	9.21	8.05	8.31	**3.43**	**2.03**
t_2	1.16	1.40	5.33	6.54	7.35	9.21	8.04	8.31	**3.31**	**2.13**
Batch 2										
t_0	1.15	1.39	5.36	6.53	7.35	9.19	8.05	8.29	**4.62**	**2.07**
t_1	1.15	1.41	5.36	6.53	7.35	9.19	8.05	8.29	**4.62**	**2.10**
t_2	1.19	1.43	5.35	6.52	7.34	9.19	8.04	8.29	**4.29**	**2.11**
Batch 3										
t_0	1.16	1.41	5.34	6.53	7.35	9.20	8.05	8.29	**4.71**	**2.02**
t_1	1.17	1.38	5.34	6.52	7.35	9.19	8.04	8.21	**4.76**	**2.03**
t_2	1.17	1.38	5.34	6.52	7.34	9.18	8.04	8.28	**4.68**	**1.99**

2.3.3. Repeatability

**Injection**	%Area under curve								
	$^{68}Ga^{3+}$ peak 1	$^{68}Ga^{3+}$ peak 2	^{68}Ga-impurity 1	^{68}Ga-impurity 2	^{68}Ga-impurity 3	^{68}Ga-impurity 4	^{68}Ga-PSMA isomer 1	^{68}Ga-PSMA isomer 2	RCP (^{68}Ga-PSMA isomer 1 + 2)
1	1.15	1.39	5.36	6.53	7.35	9.19	8.05	8.29	85.14
2	1.15	1.41	5.36	6.55	7.35	9.19	8.05	8.29	85.22
3	1.19	1.43	5.35	6.52	7.34	9.19	8.04	8.23	82.27
4	1.15	1.38	5.35	6.53	7.36	9.19	8.04	8.29	82.86
5	1.18	1.38	5.35	6.53	7.35	9.20	8.04	8.29	82.37
6	1.15	1.40	5.35	6.53	7.38	9.18	8.04	8.29	82.27
Mean	1.16	1.40	5.35	6.53	7.36	9.19	8.04	8.28	83.57
%SD	0.018	0.019	0.0052	0.010	0.014	0.0063	0.0052	0.027	1.49
%CV	1.58	1.39	0.10	0.15	0.19	0.069	0.064	0.32	1.78

2.3.4. Recovery (accuracy)

	^{68}Ga corrected to decay (CPM)	Volume measured by gamma counter	^{68}Ga corrected to decay and volume (CPM)	Outlet/inlet ratios	Mean %	%SD
Column inlet 1	1 663 810	20 µL out of 20 µL	1 663 810	0.964990954	95.64	0.57
Column outlet 1	167 246	1 mL out of 9.60 mL	1 605 562			
Column inlet 2	1 694 369	20 µL out of 20 µL	1 694 369	0.951088222		
Column outlet 2	167 864	1 mL out of 9.60 mL	1 611 494			
Column inlet 3	1 690 050	20 µL out of 20 µL	1 690 050	0.953183634		
Column outlet 3	167 805	1 mL out of 9.60 mL	1 610 928			

2.3.5. Limit of quantification

• LOQ for [^{68}Ga]Ga-PSMA-11 isomer 2:

With a [^{68}Ga]Ga-PSMA-11 solution at 8 MBq/mL, the following radiochromatogram was obtained:

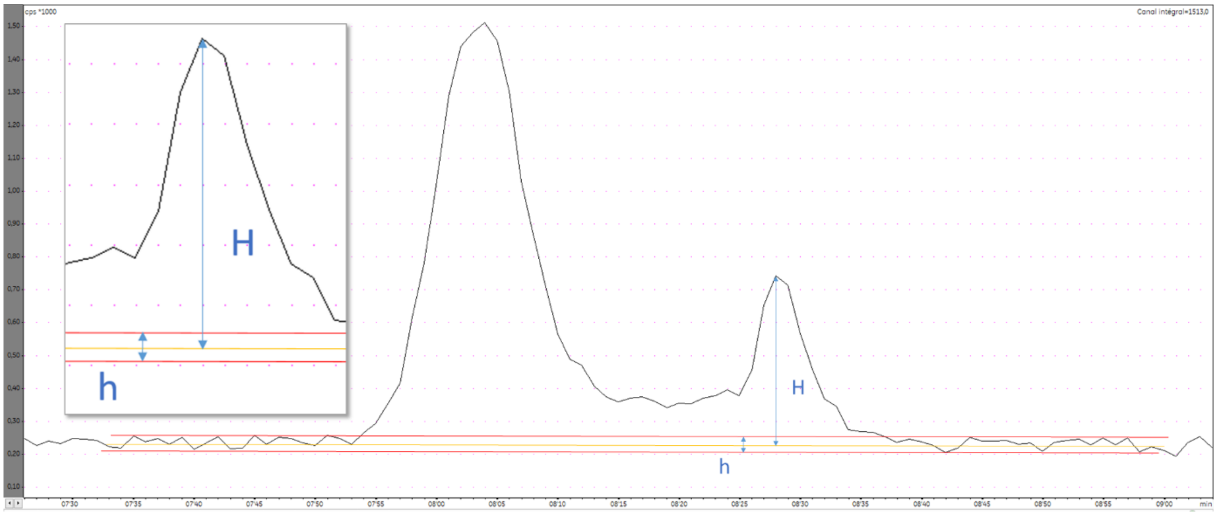

Amplitude of the background noise represented by the 2 red lines is 0.46 units (h).The height of the isomer 2 peak, from the peak apex to the extrapolated signal baseline (yellow line), is 4.78 units (H). The signal-to-noise ratio is therefore:

S/N = H/h = 4.78/0.46 = 10.39

The signal-to-noise ratio is greater than 10: the limit of quantification for isomer 2 of [^{68}Ga]Ga-PSMA-11 is therefore set at 8 MBq/mL for this radio-HPLC method.

• LOQ for [^{68}Ga]Ga^{3+} impurity:

With a [^{68}Ga]Ga^{3+} solution at 228 kBq/mL, the following radiochromatogram was obtained:

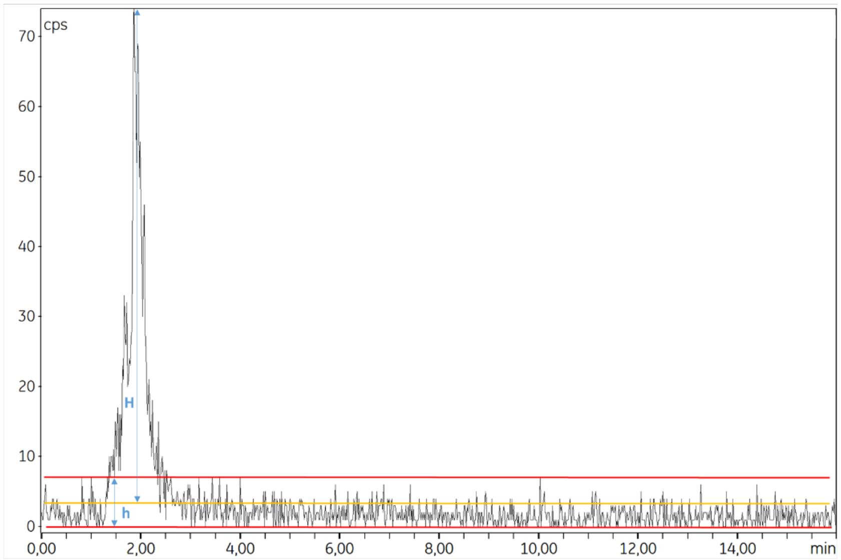

Amplitude of the background noise represented by the 2 red lines is 1.65 units (h).The height of the [68Ga]Ga^{3+} peak, from the peak apex to the extrapolated signal baseline (yellow line), is 16.74 units (H). The signal-to-noise ratio is therefore:

S/N = H/h = 16.74/1.65 = 10.15

The signal-to-noise ratio is greater than 10: the limit of quantification for [68Ga]Ga^{3+} is therefore set at 228 kBq/mL for this radio-HPLC method.

2.3.6. Limit of detection

• LOD for [68Ga]Ga-PSMA-11 isomer 2:

With a [68Ga]Ga-PSMA-11 solution at 1.05 MBq/mL, the following radiochromatogram was obtained:

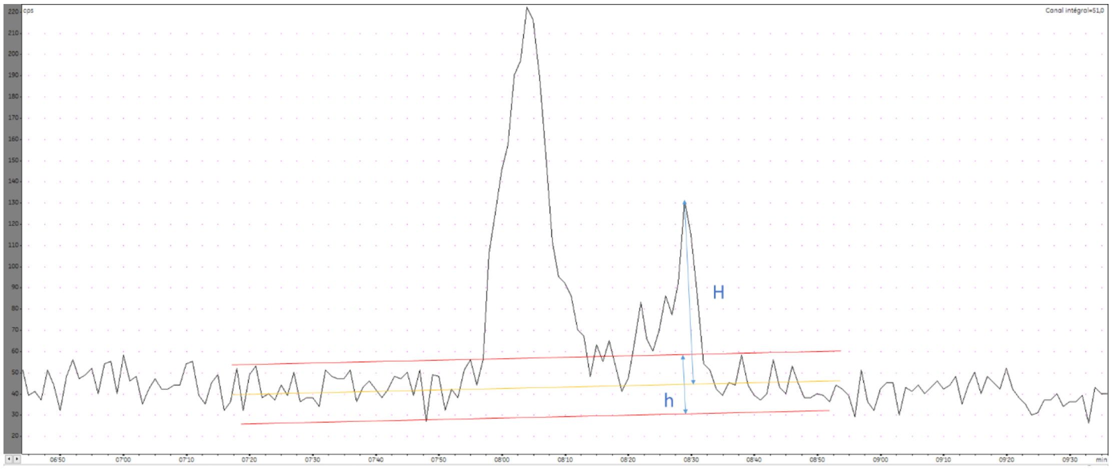

Amplitude of the background noise represented by the 2 red lines is 1.82 units (h).The height of the isomer 2 peak, from the peak apex to the extrapolated signal baseline (yellow line), is 5.67 units (H). The signal-to-noise ratio is therefore:

S/N = H/h = 5.67/1.82 = 3.11

The signal-to-noise ratio is greater than 3: the limit of detection for isomer 2 of [^{68}Ga]Ga-PSMA-11 is therefore set at 1.05 MBq/mL for this radio-HPLC method.

• LOD for [^{68}Ga]Ga^{3+} impurity:

With a [^{68}Ga]Ga^{3+} solution at 97 kBq/mL, the following radiochromatogram was obtained:

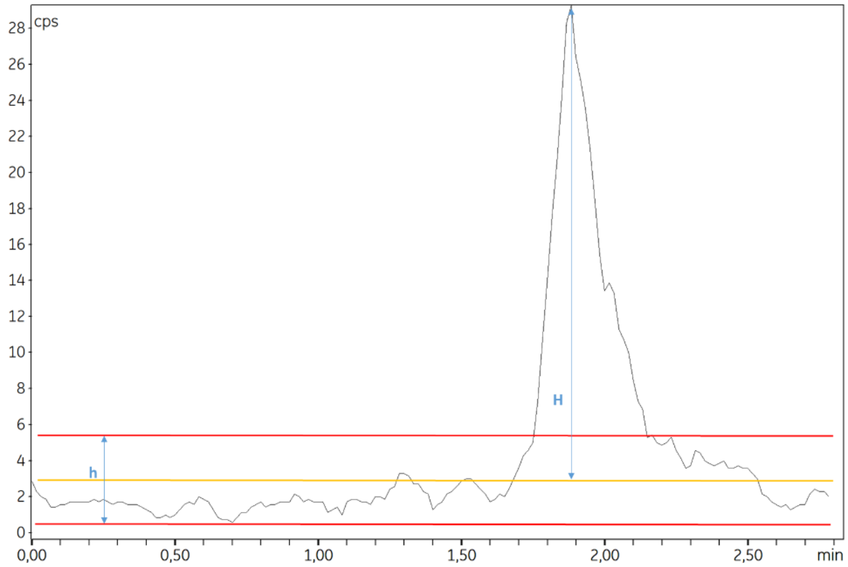

Amplitude of the background noise represented by the 2 red lines is 2.98 units (h).The height of the [^{68}Ga]Ga^{3+} peak, from the peak apex to the extrapolated signal baseline (yellow line), is 15.80 units (H). The signal-to-noise ratio is therefore:

S/N = H/h = 15.80/2.98 = 5.30

The signal-to-noise ratio is slightly greater than 3: the limit of detection for [^{68}Ga]Ga^{3+} is therefore set at 97 kBq/mL for this radio-HPLC method.

2.3.7. High-resolution test

A sample from a [^{68}Ga]Ga-PSMA-11 terminal vial was used for a high-resolution test to exclude the presence of compounds that would co-elute with [^{68}Ga]Ga-PSMA-11, and would therefore not be detected by the radio HPLC method. In this test, the initial gradient time of the radio-HPLC method (9.5 min) was doubled to improve compounds separation. All other parameters remained unchanged. The following radiochromatogram was obtained:

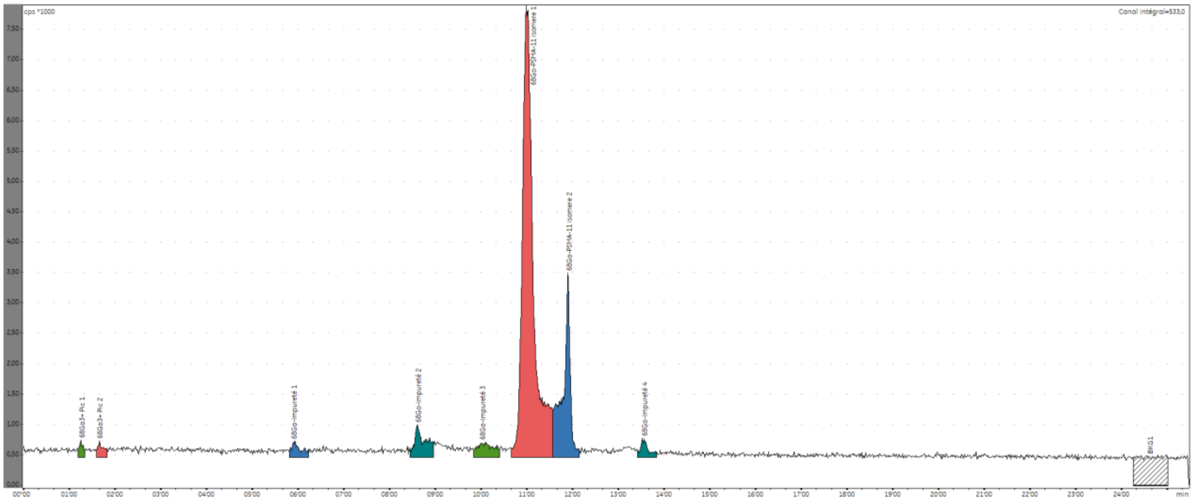

This analysis did not reveal any additional peaks: radio-HPLC discrimination of the radioactive compounds present in the final [^{68}Ga]Ga-PSMA-11 solution was therefore validated.

2.3.8. Chemical identity determined by HPLC

Substance	Retention time (min)			Mean	%SD
	Assay 1	Assay 2	Assay 3		
**UV detection**					
[^{69}Ga]Ga-PSMA-11 isomer 1	8:00	8:00	8:00	8:00	0
[^{69}Ga]Ga-PSMA-11 isomer 2	8:26	8:26	8:26	8:26	0
**Radio detection**					
[^{68}Ga]Ga-PSMA-11 isomer 1	8:05	8:05	8:04	8:05	0.12
[^{68}Ga]Ga-PSMA-11 isomer 2	8:29	8:29	8:29	8:29	0

Representative chromatogram of HPLC analysis of [^{69}Ga]Ga-PSMA-11 (detection at 220 and 280 nm):

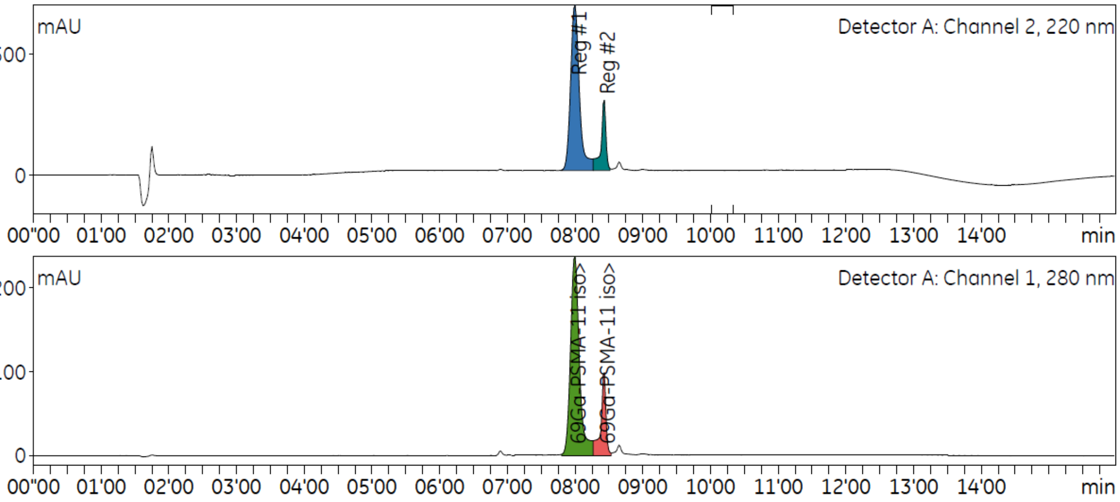

2.4. Radio-TLC methods validation

2.4.1. Radio-TLC spectrum of $^{68}Ga^{3+}$

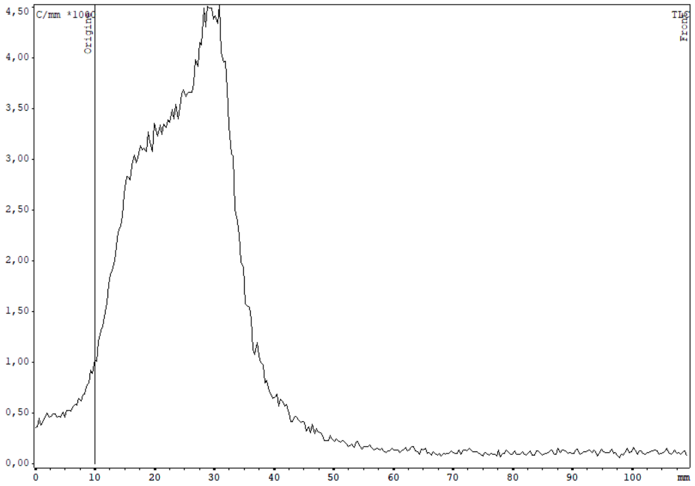

2.4.2. Radio-TLC spectrum of [^{68}Ga]gallium colloids

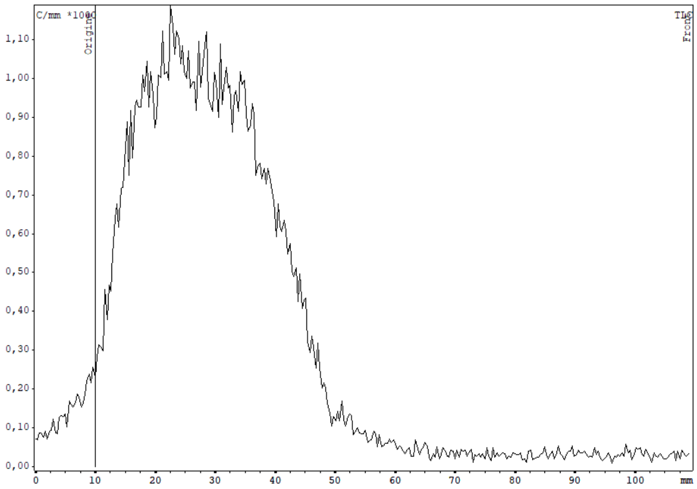

2.4.3. Radio-TLC spectrum of [^{68}Ga]Ga-PSMA-11

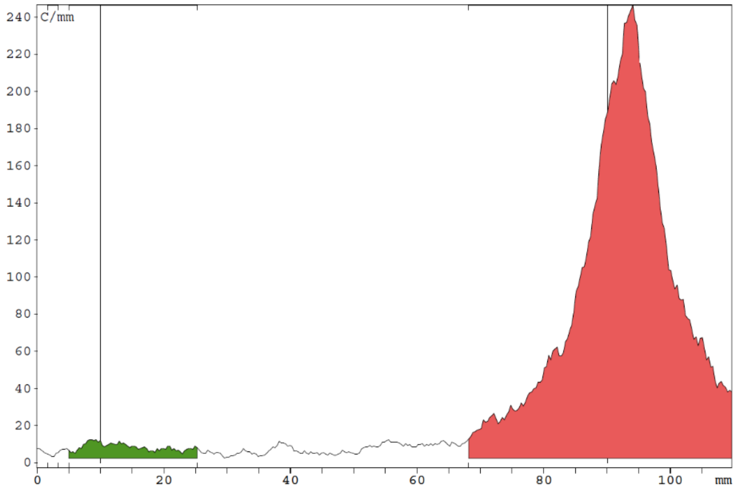

2.4.4. Radio-TLC spectrum of [^{68}Ga]Ga-PSMA-11 + $^{68}Ga^{3+}$ mixture

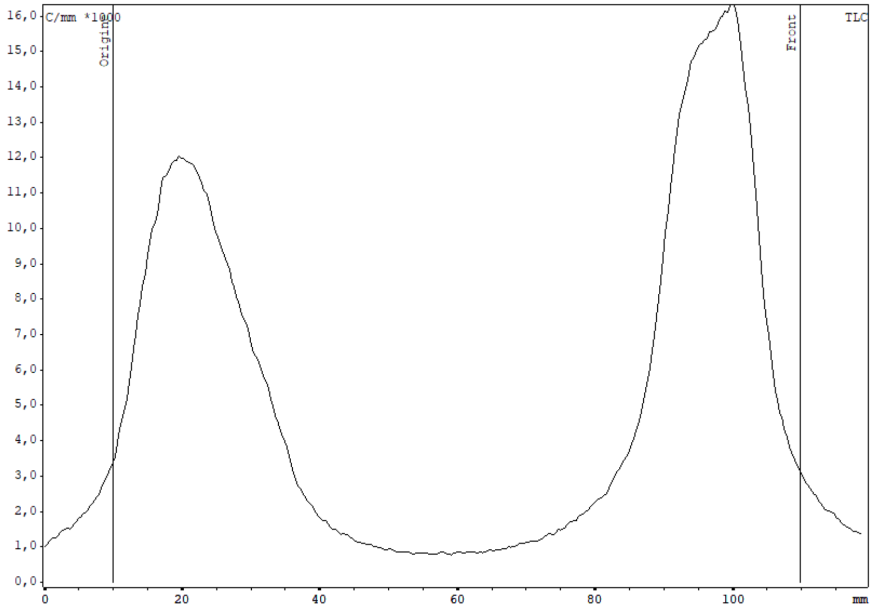

2.4.5. Radio-TLC spectrum of [^{68}Ga]Ga-PSMA-11 + [^{68}Ga]gallium colloids mixture

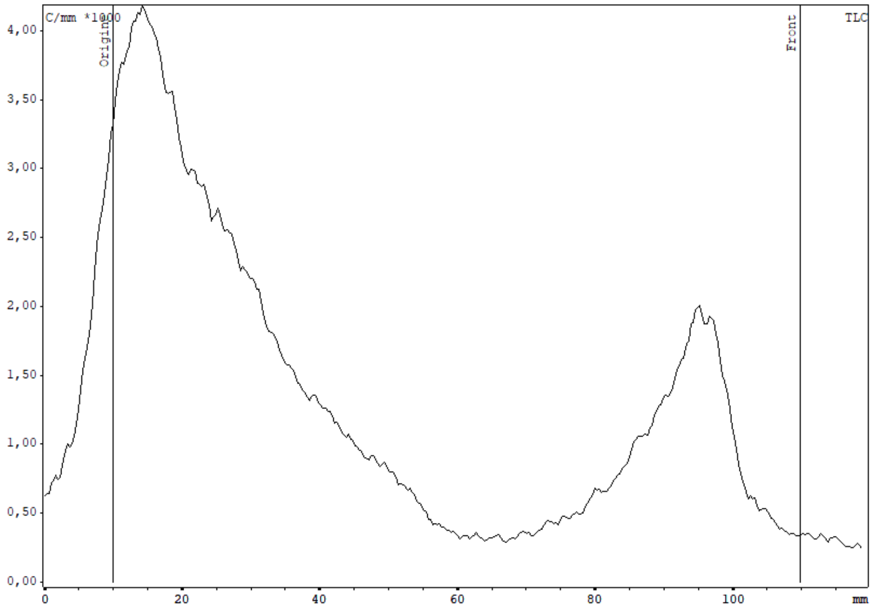

2.4.6. LOQ of ^{68}Ga impurities in radio-TLC

Analysis of a TLC plate with 5.56 kBq of [^{68}Ga]Ga-PSMA-11 final product gave the following spectrum:

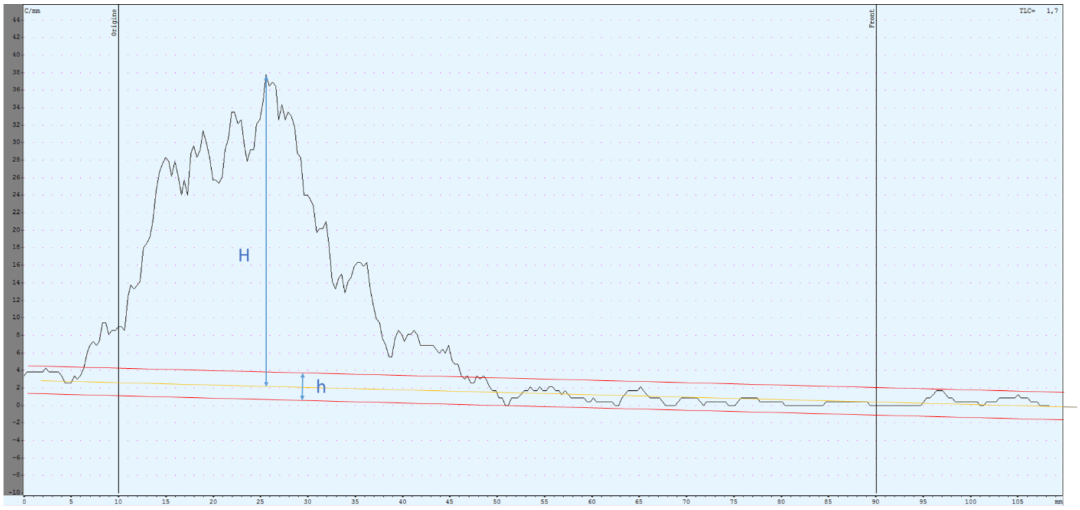

Amplitude of the background noise represented by the 2 red lines is 0.87 units (h).The height of the ^{68}Ga impurities peak, from the peak apex to the extrapolated signal baseline (yellow line), is 9.93 units (H). The signal-to-noise ratio is therefore:

S/N = H/h = 9.93/0.87 = 11.4

The signal-to-noise ratio is greater than 10: the limit of quantification of the impurity peak is therefore set at 5.65 kBq for the radio-TLC method. The minimum volume to be deposited on the TLC plate (for a preparation with the lowest acceptable activity volume, i.e. 15 MBq/mL, of which $^{68}Ga^{3+}$ represents 5% of the total activity) is therefore of the order of 7.6 µL.

3. Radiochemical stability of the validation batches

RCP (TLC)	Time of measurement after radiolabeling (h)				
	0.50	1.00	2.00	3.00	4.00
Batch 1	95.68	96.59	97.72	98.14	98.30
Batch 2	97.71	97.83	97.45	95.28	97.72
Batch 3	97.76	99.72	98.72	98.31	99.24
%SD	1.19	1.58	0.67	1.70	0.77
Mean	97.05	98.05	97.96	97.24	98.42
RCP (HPLC)	0.50	1.00	2.00	3.00	4.00
Batch 1	95.55	95.57	96.10	92.71	94.10
Batch 2	91.15	91.92	91.06	91.00	91.05
Batch 3	91.50	91.21	91.25	91.16	91.09
%SD	2.45	2.34	2.86	0.94	1.75
Mean	92.73	92.90	92.80	91.62	92.08

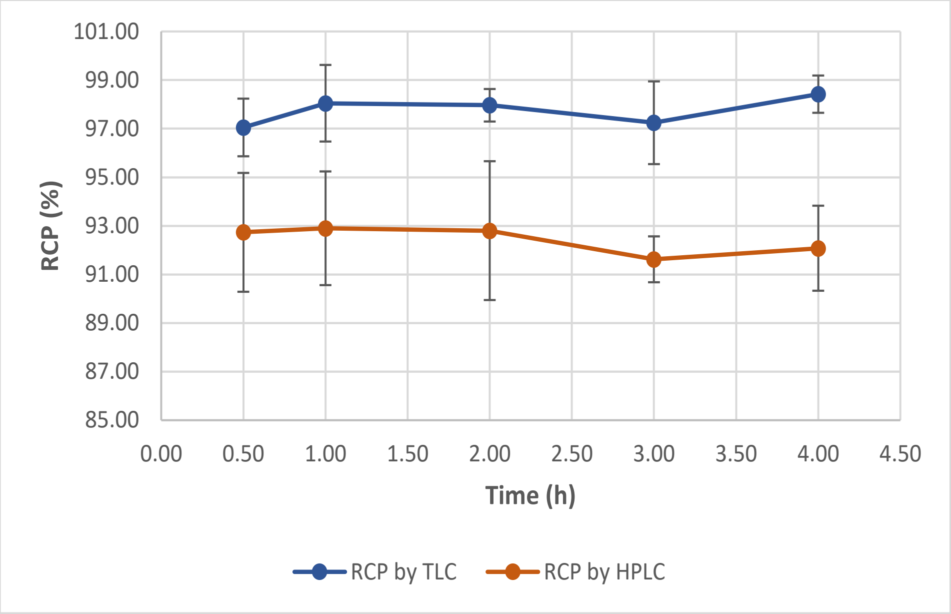

4. Adsorption of [^{68}Ga]Ga-PSMA-11 in the product vial

		Total preparation	Sampling t_0	Sampling t_1	Sampling t_2	Sampling t_3	Sampling t_4	Mean	Standard deviation	%CV
Validation batch 1	Activity (MBq)	721.00	70.24	31.77	19.42	10.25	4.20			
	Volume (mL)	10.10	1.00	1.00	1.00	1.00	1.00			
	Time (hh:mm)	8:58	9:07	10:09	11:00	12:03	13:27			
	Volume activity (MBq/mL)	71.39	70.24	31.77	19.42	10.25	4.20			
	Decay-corrected volume activity (MBq/mL)	71.39	70.24	59.93	61.74	62.09	60.07	62.81	2.97	4.73
Validation batch 2	Activity (MBq)	610.00	52.28	22.77	13.54	7.19	3.39			
	Volume (mL)	10.10	1.00	1.00	1.00	1.00	1.00			
	Time (hh:mm)	08:48	09:00	10:10	11:03	12:06	13:21			
	Volume activity (MBq/mL)	60.40	52.28	22.77	13.54	7.19	3.39			
	Decay-corrected volume activity (MBq/mL)	60.40	59.11	52.71	53.94	54.55	55.51	55.16	1.72	3.11
Validation batch 3	Activity (MBq)	651.00	62.20	29.27	14.63	7.92	3.82			
	Volume (mL)	10.10	1.00	1.00	1.00	1.00	1.00			
	Time (hh:mm)	08:39	08:47	09:48	10:57	11:56	13:07			
	Volume activity (MBq/mL)	64.46	62.20	29.27	14.63	7.92	3.82			
	Decay-corrected volume activity (MBq/mL)	64.46	67.51	59.33	60.09	58.88	59.44	61.62	2.91	4.72

5. Representative spectra for [^{68}Ga]Ga-PSMA-617 and [^{68}Ga]Ga-PSMA-I&T

5.1. [^{68}Ga]Ga-PSMA-617 radio-TLC spectrum

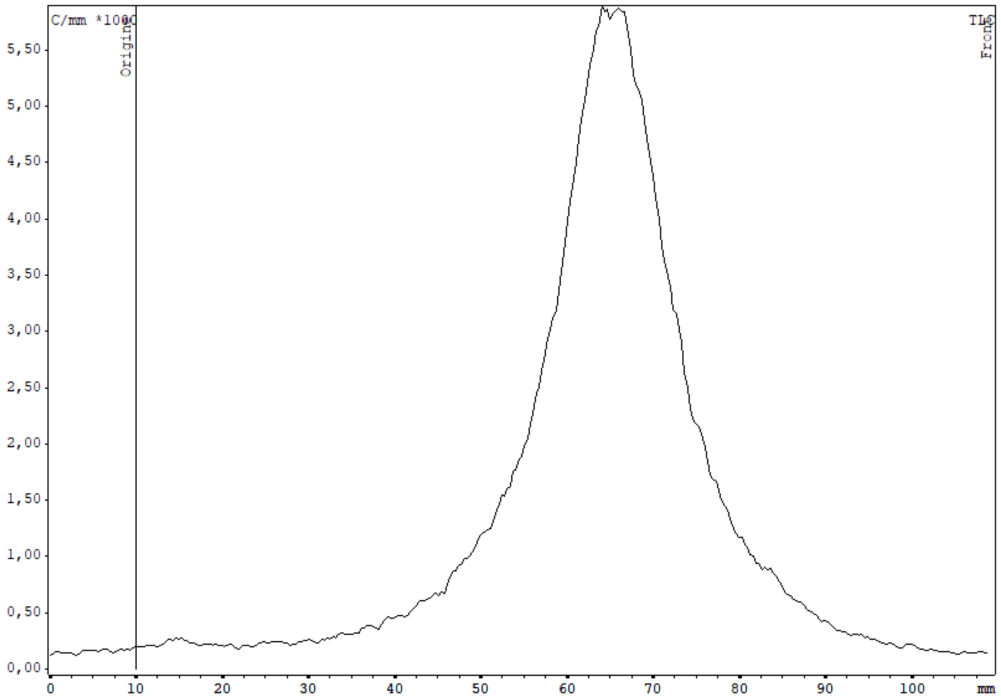

5.2. [^{68}Ga]Ga-PSMA-617 radio-HPLC spectrum

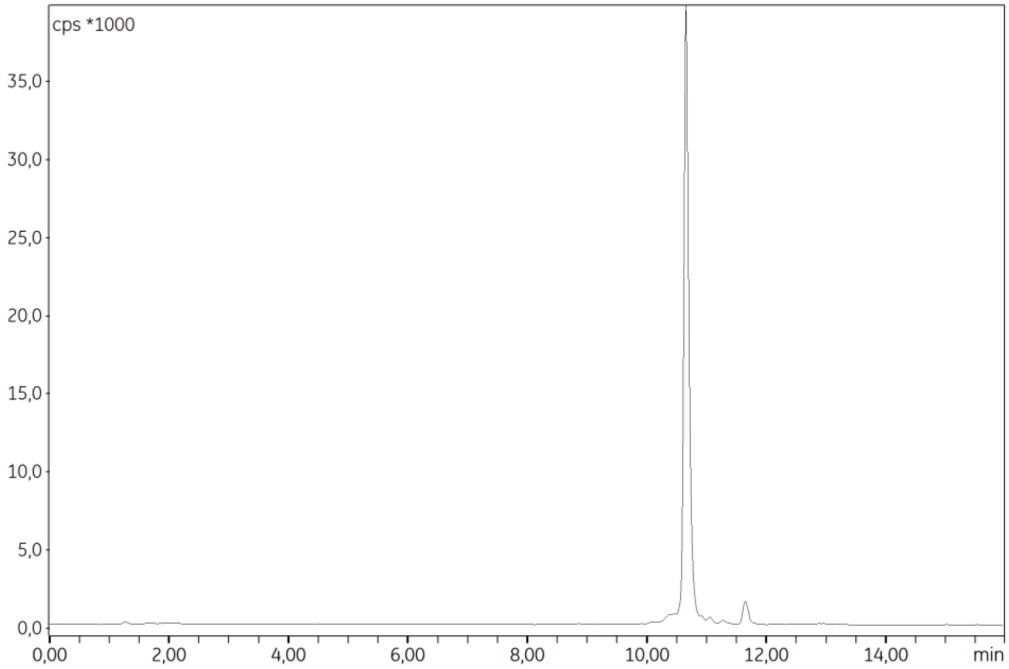

5.3. [^{68}Ga]Ga-PSMA-I&T radio-TLC spectrum

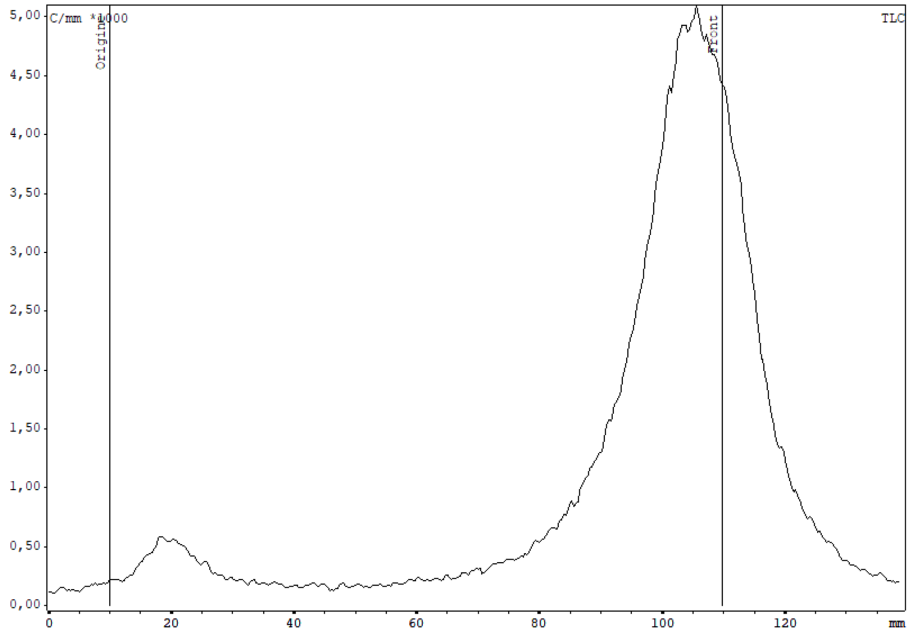

5.4. [^{68}Ga]Ga-PSMA-I&T radio-HPLC spectrum

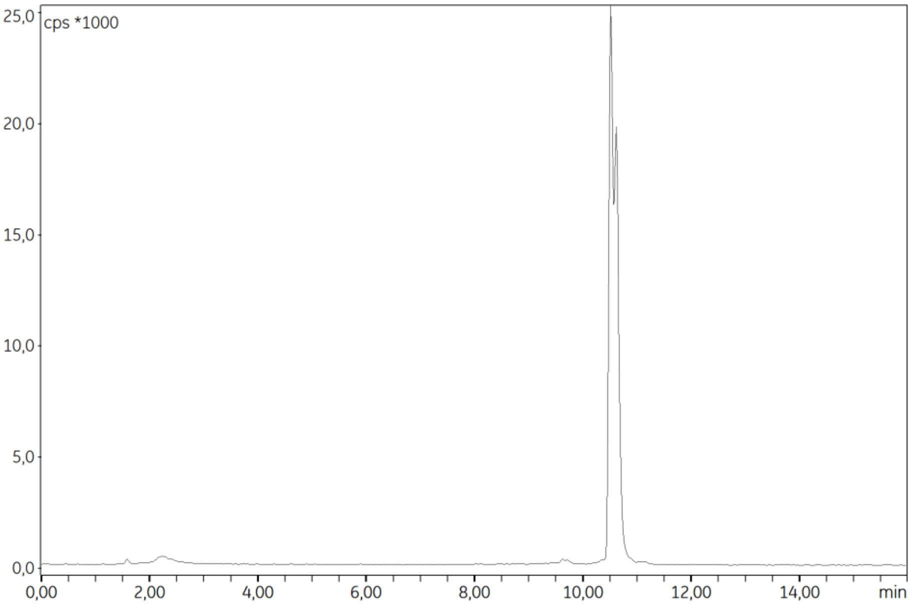

6. Determination of HEPES content in [^{68}Ga]Ga-PSMA-I&T final preparation

The determination of HEPES residual content in [^{68}Ga]Ga-PSMA-I&T final preparation was performed according to the semi-quantitative TLC method proposed by Ph. Eur. in the GALLIUM (^{68}Ga) PSMA-11 INJECTION monography. Reference solution (R) concentration was 49.5 µg/mL, equivalent to 500 µg HEPES in a total preparation volume of 10.1 mL. Test solution (A) was a sample of the [^{68}Ga]Ga-PSMA-I&T final preparation. 40 µL of each solution was deposited on the TLC plate using capillary tubes. After elution in a acetonitrile/methanol/water mixture (75:15:10) and revelation in an iodine vapor chamber, the following plate was obtained:

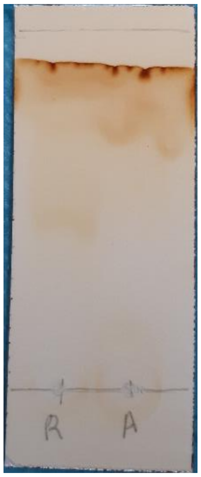

On the migration path of the teste solution (A), no spot that could be more intense than the corresponding spot in the reference solution path (R) could be identified. This confirms that the residual amount of HEPES in the [^{68}Ga]Ga-PSMA-I&T final preparation is below the concentration limit set by Ph. Eur.
